# Football (Soccer) refereeing and cardiovascular health: A scoping review

**DOI:** 10.1371/journal.pone.0346360

**Published:** 2026-04-02

**Authors:** Toby M Plasto, Geoffrey H Tofler, Tom Buckley

**Affiliations:** 1 School of Medicine and Health, The University of Sydney, Sydney, New South Wales, Australia; 2 St Vincent’s Hospital, Darlinghurst, New South Wales, Australia; 3 Department of Cardiology, Royal North Shore Hospital, St Leonards, New South Wales, Australia; Portugal Football School, Portuguese Football Federation, PORTUGAL

## Abstract

**Background:**

Physical and psychological stress can precipitate Acute Coronary Syndrome (ACS) events. Football (soccer) is a popular sport globally, with referees covering significant distances, often at high-intensity during a match, and facing potentially intense psychological stress from high-stakes decision-making and potential verbal abuse from players and spectators. The aim of this scoping review was to identify what is known about the cardiovascular health and risk of acute cardiac events in referees during football matches.

**Methods:**

A librarian-assisted search of six databases was completed, with publications written in English or with English translation available included. The PRISMA checklist was utilised and data were extracted from publications to form both descriptive and thematic analyses.

**Results:**

Analysis of the publications identified five key themes: 1) the cardiovascular risk factors profile of football referees (prevalence of hypertension, dyslipidaemia and diabetes), 2) psychological stress and abuse experienced by referees (both verbal and physical abuse are reported at all levels of football, contributing to stress, anxiety, and anger), 3) physical intensity and activity levels of football referees (distance covered per match), 4) physiological aspects of football refereeing and 5) cardiac events on the football field (referees are rarely involved in providing CPR).

**Conclusions:**

Evidence indicates that football referees face significant cardiovascular demands and psychological stress, along with a vulnerability to physical abuse, yet there is a lack of research on educational interventions to promote their cardiovascular health. Additionally, referees may act as first responders during ACS events, highlighting the necessity for them to be knowledgeable about relevant symptoms and trained for effective response.

## Introduction

Aerobic exercise is typically thought of as beneficial for cardiovascular health, muscle strength and mental health [1]. However, a paradox exists where an increase in myocardial infarction (MI) and sudden cardiac death is seen occasionally in those exercising or who have recently completed exercise [2]. While these events are rare, particularly during football (soccer) matches, they are often widely publicised, such as in the case of Christian Eriksen, a Danish professional football player who collapsed on the pitch during the EURO 2020 competition and was resuscitated on the field [3]. Several anecdotal reports in the print media highlight cardiac events in football referees too, including an international-level official requiring a coronary stent after suffering an MI at home following a training session [4], a Bolivian referee dying in 2019 after suffering an MI on the field [5] and a referee’s life being saved in an American College football match with the aid of a defibrillator [6].

Football referees not only require sufficient level of cardiorespiratory fitness but are required to be mentally focussed for the duration of the match, where split second decisions can impact on the outcome. This decision making can be further complicated by the fact that players may exaggerate their response to draw support from the referee. Additionally, amateur football can be seen by some players to be a stress outlet, potentially releasing pent-up emotions from work and family life [7]. All of these factors make refereeing a football match potentially a complex, stressful and challenging task. Since emotional stressors and strenuous exertion are known to precipitate Acute Coronary Syndromes (ACS) [8–10], these factors combined suggest that football referees are potentially at increased risk of experiencing ACS events themselves during a game and may also be required as first responders for other participants of a football match.

A scoping review was conducted to summarise and map the available peer-reviewed literature related to cardiovascular risk factors, and risk of ACS in football referees. The findings inform a program of research investigating ways to potentially support referee cardiovascular health.

## Methods

### Search strategy

The scoping review was guided by the recommended methodology of Arksey and O’Malley [11] and expanded on by Levac and colleagues [12]. The Preferred Reporting Items for Systematic Reviews and Meta-Analysis (PRIMSA) and its extension for Scoping Reviews (PRISMA-ScR) checklist was used (S1 Fig). No ethical approval was required for this review.

Stage 1: Identify the research question/s

Research questions were formulated: “What is known about cardiovascular risk factors in football referees?” and “What data or evidence exists regarding cardiovascular events in football referees?”.

Stage 2: Identifying relevant studies

Studies were included if they met the following inclusion criteria:

- Referees that officiate football games of any age group, sex or geographical location
- Published in English in peer-reviewed journal or with English translations available
- Evaluated potential cardiovascular risk factors or cardiovascular events on the pitch in which referees were involved

Exclusion criteria:

- Opinion pieces/opinions, magazine articles, case reports, papers with no data
- Studies where the key focus was on decision-making of football referees and not the impact of decisions or cardiovascular risk factors

### Search strategies and databases

A librarian-assisted systematic search of six databases was completed on 8 June 2024, including: CINAHL, Excerpta Medica database (EMBASE), MedLine, SCOPUS, SPORTDiscuss and Web of Science, utilising Boolean operators with keywords, with consultation with a university librarian (S2 Fig). A further search was completed on 21 July 2025, with one new publication identified.

Stage 3: Study selection

Title and abstract screening were completed through the online application EndNote, with the software utilised to remove duplicates, with a manual review following this. Primary screening of titles and abstracts was completed by one reviewer (TMP), with discussion with a second reviewer (TB). Any disagreements were resolved with input from a third reviewer (GT). Full text articles were then retrieved utilising the EndNote software. Despite a thorough database search, including university librarian support, 21 full-text articles could not be found and were excluded from final analysis.

Stage 4: Charting the data

For each article, key information was extracted into a matrix table as described by Pollock and colleagues [13]. Information included: author, year, location, journal, design, objective, methods and conclusion.

Stage 5: Collating, summarising and reporting results

Articles were collated into a summarised matrix table (S1 Table) with key themes identified from the data, with inductive coding of themes as discussed by reviewers and further subcategorised based on any existing sub-themes, this was done in an iterative manner, with ongoing refinement until complete agreement between reviewers.

### Patient and public involvement

As this is a scoping review of existing data and publications, there is no specific patient or public involvement.

## Results

### Findings and patterns

**Fig 1:**
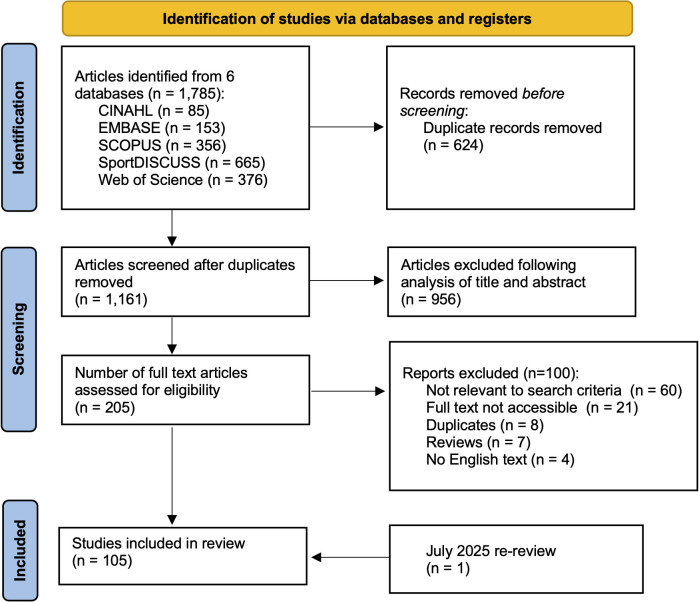
PRISMA diagram demonstrating the search method for identifying and screening articles.

**Fig 2:**
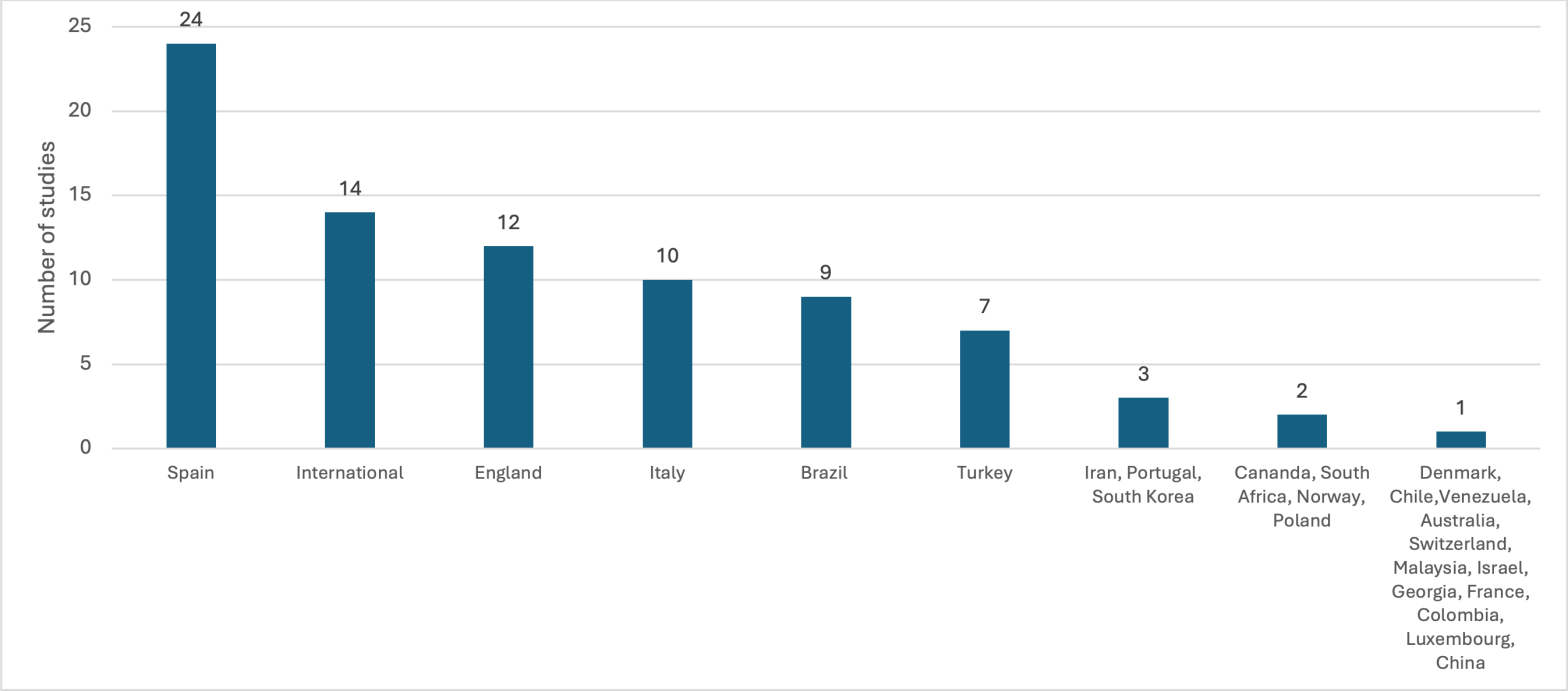
Country of origin of included studies.

**Fig 3:**
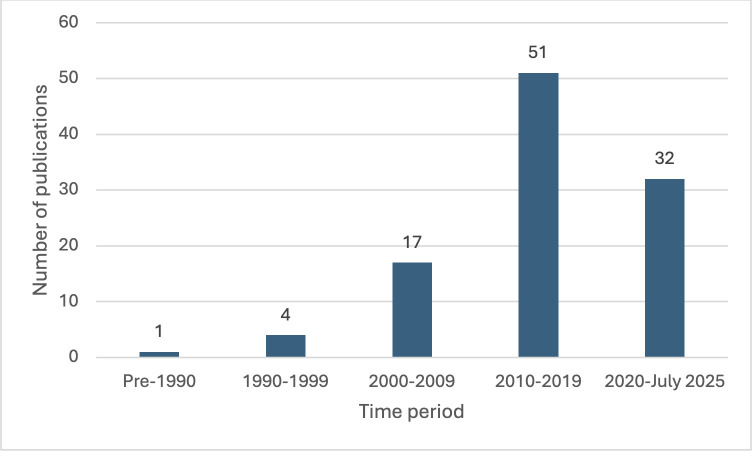
Publications by year of publication.

**Fig 4:**
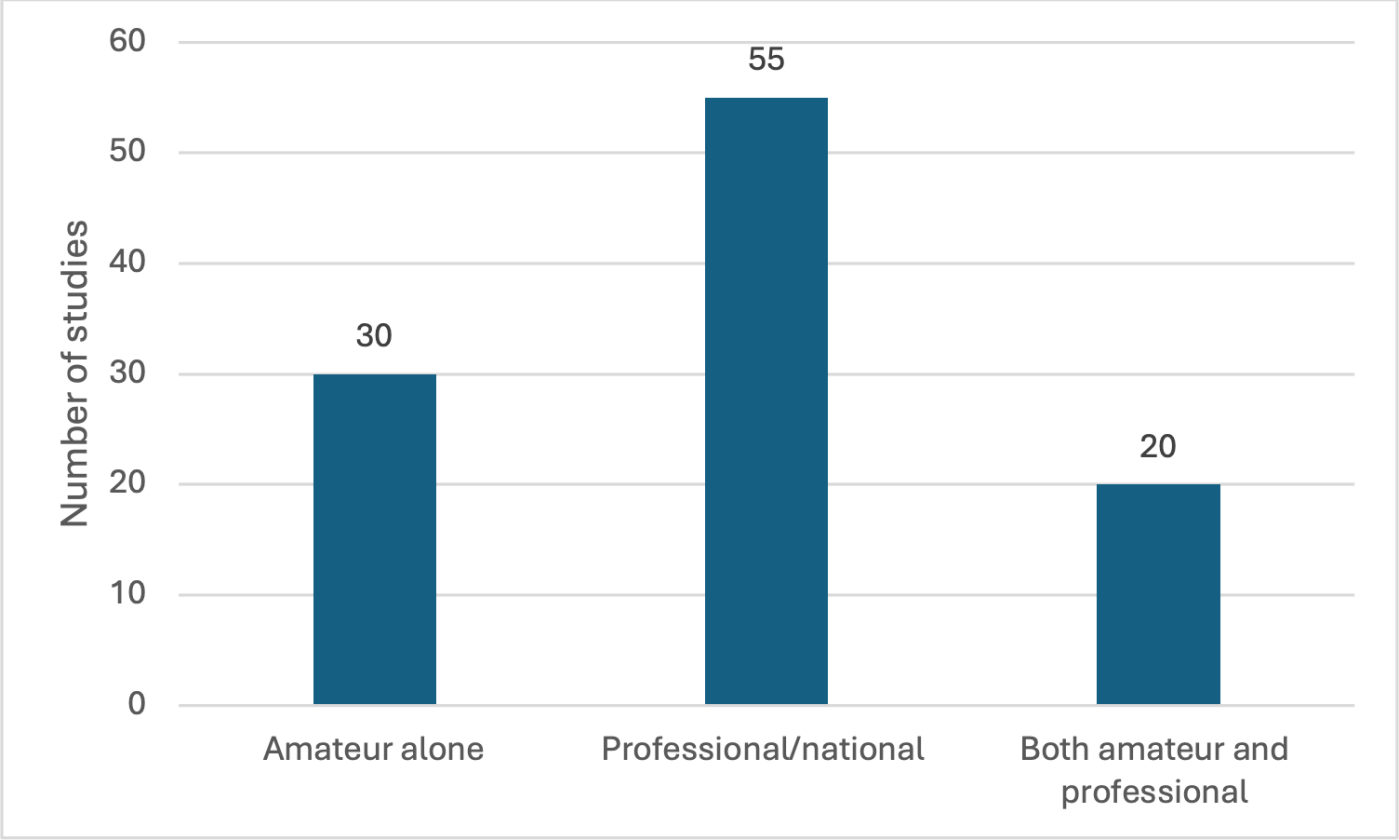
Referee cohort included in the study.

In total, 105 research study publications were included for final review, (Fig 1). The countries of included studies is presented in Fig 2, with a majority (61.0%) utilising European referees, a further 14 utilising an international group of football referees, with the remaining studies originating from a variety of countries. Five publications were published prior to 2000, with a notable increase in publications since 2010 (Fig 3). Fifty-five studies focussed on national level or professional level football referees, 30 studies evaluated amateur football referees and 20 studies included both amateur and professional referees (Fig 4).

### Key themes identified

Analysis of the publications identified five key themes in the research literature: 1) cardiovascular risk factors of football referees, 2) psychological stress experienced by referees, 3) physical activity of football referees, 4) physiological aspects of football refereeing and 5) cardiac events on the football field (Table 1).

**Table 1:** Themes and patterns from included studies.

	Study Type							Themes				
Author/s. (Year).	Case report/series	Qualitative	Case control	Cohort study	Observational	Cross-sectional	Longitudinal	CVD risk factors	Psychological stress (incl. abuse)	Physical activity	Cardiovascular parameters	Cardiac events in match
**Taylor, A. et al.** **(1988).**		x							X			
**Catterall, C. et al.** **(1993).**					x					x	x	
**Hemmings, B. & Graydon, J. (1994).**		x							x			
**Johnston, L & McNaughton L.** **(1994).**					x					x	x	
**Ladelfa, G. et al.** **(1994).**	x									x	x	
**D’Ottavio, S. & Castagna, C. (2001).**	x				x					x	x	
**D’Ottavio, S. & Castagna, C. (2001).**	x									X		
**Krustrup, P. & Bangsbo, J. (2001).**	x									x	X	
**Folkesson, P. et al.** **(2002).**		x							x			
**Castagna, C. & Abt, G.** **(2003).**	x				x					X		
**Castagna, C. et al.** **(2004).**				x						X		
**Helsen, W. & Bultynck, J. (2004).**	x				x					x	x	
**Castagna, C. et al.** **(2005).**				x	x					x	x	
**Mallo, J et al.** **(2006).**	x				x					x	x	
**Müniroglu, S.** **(2007).**	x				x					x	x	
**Fernández Vargas, G.E. et al.** **(2008).**					x					x	X	
**Galanti, G. et al.** **(2008).**				x							X	
**Gardener, P.** **(2008).**									X			
**Gencay, S.** **(2009).**		x							x			
**Krustrup, P. et al.** **(2009).**	x				x					x	x	
**Mallo, J. et al.** **(2009).**	x				x					x	x	
**Mallo, J. et al.** **(2009).**	x				x					x	x	
**Voight, M.** **(2009)**		x							x			
**Bambaeichi, E. et al.** **(2010).**	x	x			x			x	x		X	
**Weston, M. et al.** **(2010).**					x					X		
**Can, Y. et al.** **(2011).**		x							x			
**Di Salvo, V. et al.** **(2011).**				x						x		
**Lategan, L.** **(2011).**	x									x	X	
**Ruiz Caballero, J.A. et al.** **(2011).**	x				x						x	
**Barbero-Alvarez, J. et al.** **(2012).**	x				x					x	x	
**Bizzini, M. et al.** **(2012).**	x							x		x	x	
**Boullosa, D.** **(2012).**	x				x					x	x	
**Halabchi, F. et al.** **(2012).**	x									X		
**Adnan, R. et al.** **(2013).**	x									X		
**Costa, E.C. et al.** **(2013).**	x				x					x	x	
**Johansen, B.T. & Haugen, T.** **(2013).**		x							x			
**Neil, R. et al.** **(2013).**		x							X			
**Blumenstein, B. & Orbach, I.** **(2014).**	x								x	X		
**Slack, L.A. et al.** **(2014).**		x							X			
**Yanci-Irigoyen, Y.** **(2014).**							x			x	x	
**Lela Maskhulia, L. et al.** **(2015).**	x							x		x	x	
**Parsons, T. & Bairner, A.** **(2015).**		x							x			
**Pedrosa, I. & Garcia-Cueto, E.** **(2015).**		x							X			
**Slack, L.A. et al** **(2015).**							x		X			
**Castillo, D. et al.** **(2016).**	x									X		
**Castillo, D. et al.** **(2016).**	x									x	x	
**Mazaheri, R. et al.** **(2016).**						x					X	
**Pedrosa, I. & Garcia-Cueto, E.** **(2016).**		x							X			
**Rebolé, E. et al.** **(2016).**	x									x	x	
**Bozdogan, T.K. et al.** **(2017).**	x			x			x			x	x	
**Dolański, B. et al.** **(2017).**	x									x	X	
**Gianturco, L. et al.** **(2017).**	x									x	x	
**Gouttebarge, V. et al.** **(2017).**				x	x				X			
**Niederseer, D. et al.** **(2017).**	x							x		x	x	
**Sirin, Y. & Dosyilmaz, E.** **(2017).**	x								x			
**Soriano, G. et al.** **(2017).**		x							X			
**Webb, T. et al.** **(2017).**		x							x			
**Castagna, C. et al.** **(2018).**				x						x	x	
**Castillo Alvira, D. et al.** **(2018).**	x									x	x	
**Choi, Y. & Roh, J.** **(2018).**						x				x	x	
**Da Gama, D.R.N. et al.** **(2018).**						x			X			
**de Moura Simim, M.A. et al.** **(2018).**		x							X			
**de Oliveira, Ab et al,** **(2018).**		x							X			
**Gama,D.R.N.D. et al.** **(2018).**		x							X			
**Gillue, G.S. et al.** **(2018).**		x							X			
**Jorge-Soto, C. et al.** **(2018).**	x									x		x
**Kilic, O. et al.** **(2018).**				x					x	x		
**Campo, M. & Louvet, B.** **(2019).**		x							x			
**Castagna, C. et al.** **(2019).**						x				x	x	
**Chang Hwa, J.O.O. et al.** **(2019).**	x									x	X	
**da Silva, V.L. et al.** **(2019).**						x				x	X	
**Fuente, D. et al.** **(2019).**		x							X			
**Hong, E. et al.** **(2019).**		x							X			
**Santos-Silva, P.R. et al.** **(2019).**	x									x	x	
**Aguirre-Loaiza, H. et al.** **(2020).**						x			X			
**Senecal, I. et al.** **(2020).**	x									x	x	
**Webb, T. et al.** **(2020).**		x							X			
**Aguilar, J.L. et al.** **(2021).**						x			X			
**Castillo-Rodriguez, A. et al.** **(2021).**	x								x		x	
**Devís-Devís, J. et al.** **(2021).**		x							X			
**Gorczynski, P. & Webb, T.** **(2021).**		x							X			
**Marchante, A. & Cervigon, R.** **(2021).**						x			x	x	x	
**Martín-Sánchez, M.L. et al.** **(2021).**	x								X			
**Moreno-Perez, V. et al.** **(2021).**				x						x	x	
**Orviz-Martinez, N. et al.** **(2021).**		x							X			
**Özdamar, E. et al.** **(2021).**	x								x		X	
**Rebelo-Gonçalves, R. et al** **(2021).**	x									x	x	
**Urhausen, A. et al.** **(2022).**	x									x	x	
**Arjona, C.M. et al.** **(2022).**	x								x	x	x	
**Castillo-Rodríguez, A. et al.** **(2022).**				x					x	x	x	
**Dawson, P. et al.** **(2022).**		x							X			
**Fernandez, X.E. et al.** **(2022).**						x			x			
**Ferreira, M. et al.** **(2022).**						x		x	x	x	x	
**Gorczynsk, P. & Thelwel, R.l** **(2022).**						x			X			
**Liu, Z.Y. et al.** **(2022).**		x							X			
**Marin-Montin, J. & Bianchi, P.** **(2022).**		x							X			
**Martin-Sanchez, M.L. et al.** **(2022).**	x									x	x	
**Nogueira, D. et al** **(2022).**		x							X			
**Ozaeta, E. et al.** **(2022).**	x									x	x	
**Potrac, P.A. et al.** **(2022).**		x							X			
**Sánchez, J. M. et al** **(2022).**	x									x	x	
**Wesolowska, J. et al.** **(2022).**				x				x	x		X	
**da Silva, J.F. et al.** **(2022).**	x					x				X	x	
**Lima, S.Y. et al.** **(2022).**	x								x			
**Martinez-Torremocha, G. et al.** **(2022).**	x					x			x	x	x	

### Cardiovascular risk factors in football referees

The prevalence of cardiovascular risk factors in different football referee populations have been reported in five studies, reporting on risk factors such as hypertension, dyslipidaemia and tobacco smoking [14–18], all without control comparisons. In referees selected for the 2010 and 2014 FIFA World Cup, cardiovascular risk factors reported included dyslipidaemia (prevalence 2.2% (n = 2) in 2010 cohort vs 16.0% (n = 25) in 2014 cohort) and hypertension (prevalence 1.1% (n = 1) in the 2010 cohort vs 4,5% (n = 7) in 2014 cohort) [16,17].

One study compared referees with a non-referee population, reporting lower Body Mass Index (BMI), less truncal and visceral adipose tissue, significantly lower levels of hypertension and fewer tobacco smokers in the referee group, although this study was not age-matched [19]. In addition, three studies reported that elite level football referees had similar heart structure to football players when assessed by echocardiography with regard to left ventricular (LV) mass, LV wall thickness and LV cavity dimension [20–22].

### Stress Testing in football referees

Studies of electrocardiogram (ECG) testing in football referees from all levels, including World Cup referees, indicate a low prevalence of pathological changes, with occasional instances of ST segment depression and T-wave inversion, which did not correlate with significant cardiovascular disease on follow up [15,17,18]. Overall, these results indicate that while some referees may demonstrate non-specific ECG findings, the majority demonstrate normal cardiac function.

### Psychological stress reported by football referees

Psychological stress is a commonly experienced emotion among football referees, particularly due to fear of making a mistake [23–26], lack of formal recognition for their efforts [26,27] and fear of physical or verbal abuse [23–30]. Stress from factors external to football, such as family support, travel for matches and non-referee occupational stress also impact the emotionality and functionality of referees [7,31–34]. Three studies have also reported similar levels of perceived stress during a match between the referee and their assistant referees [35–37].

More experienced football referees generally experience a greater prevalence of positive psychological emotions and fewer negative [38–47]. However, a study involving elite Danish officials found that they experience higher anxiety levels than those refereeing games at a lower levels [48]. Additionally, referees <38 years of age exhibited greater anxiety, depression, burnout and stress than their older counterparts [41,42,49,50]. Notable one study revealed that negative emotions often surface at the start of a match, with initial feelings of happiness and excitement giving way to anxiety at kick-off [51]. Furthermore, single season studies of professional referees in Europe documented an increase in anxiety, depression, sleep disturbance and eating disorders alongside a decrease in self-confidence by the end of the season compared with pre-season testing [52,53]. Mental health conditions such as anxiety/depression (up to 43% prevalence), eating disorders and alcohol misuse (20% prevalence) [52] have been reported in European football referees [54], with increased prevalence compared with the general population.

Psychological burnout has been reported at all levels of refereeing [41], including in Spanish (prevalence 2.4%) [55] and Turkish professional referees (mean score 24.5 on Pines and Aronson’s (1988) burnout scale, indicating low-moderate levels of burnout) [56]. Female referees have been noted to have higher prevalence of burnout than their male colleagues [57–59] and centre field referees reported higher incidence of burnout than assistant referees [56,60].

### Abuse faced by football referees

In Spain, football accounts for 93–98% of violent incidents in sports, with referees being the targets in 40–50% of these occurrences, as indicated by a review from the Spanish Department of Sport [61]. Notably, the total number of reported incidents decreased by nearly 50% over the decade from 2006 to 2016; however, the authors did not provide an explanation for this decline.

A study of 4,367 French and Dutch officials found that 68.1% of French and 51% of Dutch referees had experienced some form of verbal abuse, while physical abuse was reported in 17% of the French cohort and 15% of the Dutch cohort [62]. These participants were recruited via emails from their national refereeing department and abuse was assessed via self-reported questionnaires. The findings on physical abuse are consistent with a study which reported that 15% of surveyed Swedish referees had been physically abused in their role [58]. Additionally, it was noted that 22% of surveyed French referees and 12% of Dutch referees were considering quitting refereeing in the next 12 months due to the abuse experienced [63].

Abuse is a key reason why football referees cease officiating, with former English referees from all levels of the game identifying the main reason for leaving refereeing as due to the emotional stressors and abuse faced from other participants in the sport [64], which is reinforced in a study of Canadian officials [65]. Campaigns to ameliorate the stressors faced by football referees have struggled to gain traction or have meaningful outcomes [66–68].

### Physical activity of football referees

Professional football referees cover a substantial distance during matches, averaging between 9–14 km, while non-elite officials cover 6.7 to 11 km; female referees average around 10 km [69–88]. Assistant referees typically cover 5.8 to 6.8 km, engaging in more walking and a higher frequency of sprints compared to centre referees [73,80]. Elite referees cover more distance than amateur ones [89], with a significant portion (>45%) of their movement occurring at speeds exceeding 13.1 km/hr [70,71,80,84,90]. Referees utilize various movement patterns, including running, walking, and moving backwards to optimize their positioning [81,90,91], while assistant referees may cover up to 30% of their distance side-stepping [88]. Research on distance covered in each half is mixed, with some studies showing no significant differences between halves [73,90,92,93], while others indicate a decrease in overall distance or medium-intensity activity during the second half [78,86,94–97].

### Cardiovascular intensity whilst refereeing a football match

Both amateur and elite football referees operate at a high level of cardiovascular intensity for significant periods of a match [98], based on maximum HR (HRmax) calculations, often spending >80% of a match at >85% of estimated HRmax [99–102]. This has been reported in referees from many countries including Spain [103], England [86], Italy [69,92,96,104,105], Brazil [1,106], Australia [81], South Africa [107], Denmark [79], Iran [108] and in European international-level referees [109–111]. In addition to heart rate, lactate levels were noted to be elevated in match officials post-match compared with pre-match [112,113]. Assistant referees appear to spend less time during a match at high levels of cardiovascular intensity compared to central referees, with only 20% of match time spent with HRmax > 85%, with longer periods of standing [114–116].

There are fewer studies focussed on female referees, however it appears from studies that they have a similar cardiovascular response during a match to male referees [99] but fewer sprints per match [88,117,118].

### Cardiac events on the football field

An analysis of a five-year period of the International Federation of Association Football (FIFA) sudden death registry (FIFA-SDR) from 2014–2018 identified that there were 617 cases of reported sudden deaths in players, with 95% occurring at the amateur level [119]. 76% of cases reported information about cardiopulmonary resuscitation (CPR) efforts, with prompt initiation in 68% of cases and a noted increase in survival when initiated by a CPR-trained person, with referees listed as participating in the CPR efforts in ~5% of player cases [119]. No studies were identified in the literature reporting the incidence of cardiac events in referees during or after football games.

CPR competency in Basic Life Support (BLS) and Automatic External Defibrillator (AED) use in football referees was reported in one study [120]. In this study of a cohort of amateur referees, one-third of the sample knew what an AED was but only 8% knew how to use it. Following a brief education intervention that included CPR practice, all participants achieved 70% or higher CPR quality scores and were determined to be able to use the AED properly (54.2% without any incidents) [120].

## Discussion

A total of 105 studies were identified that focussed on cardiovascular events during matches, potential cardiovascular risk factors, physical activity and psychological stress associated with football refereeing. A majority of studies were case reports (n = 31), with qualitative (n = 30) and cross-sectional (n = 11) studies making up the other large portion. This area of research has begun to expand with 83 peer-reviewed publications since 2010, with the largest population of studied referees being from Spain (n = 24). Importantly given that football is known as “The World Game” this review identified that refereeing cohorts from across the world have been studied providing a breadth of populations for analysis. A majority of publications were case reports and/or observational studies.

This review identified five main themes regarding football referees, cardiovascular risk factors and exposure to potential ACS events which were: 1) cardiovascular risk factors, 2) psychological stress experienced, 3) physical activity, 4) physiological aspects of football refereeing and 5) cardiac events on the football field.

### Football referee responses to potential cardiac events

This review identified that football referees were involved in CPR when required during a match in ~5% of cases [119], although data were available in only one publication. In one study, referees knowledge was limited on CPR, however with a short education session, they were able to attain competency in CPR [120]. The FIFA-SDR database indicated prompt CPR as being beneficial to the outcomes of players who suffered from ACS events, with referees often close to players on the field due to the nature of their role [119]. According to the Laws of The Game, every formally sanctioned match should be officiated by at least one registered referee [121], providing an avenue for improvement with respect to upskilling football referees at all levels regarding BLS and CPR. This is particularly important at the amateur level, where it is unlikely there will be a team physiotherapist, medical professional or paramedic crew at the match, as is often the case at professional and semi-professional level matches.

Although media reports exist of cardiac events in football referees [4–6], the review highlighted no peer reviewed publications or central repository of information related to referees. This is a significant area for improvement at the international level in order to accurately profile the number of ACS events and sudden deaths noted in football referees.

### Cardiovascular risk factors in football referees

Referees selected for the 2010 and 2014 FIFA World Cup demonstrated prevalence of cardiovascular risk factors including dyslipidaemia and hypertension [16,17], however no studies exist comparing referees with an age-matched population. While cardiovascular risk factors have been investigated in cohorts of elite match officials (n = 4), especially focussing on biochemical-related risks, fewer (n = 2) studies focus on the amateur referee cohort, who are larger in size and likely to have higher risk factors when compared with their elite referee colleagues, who have been reported to have similar fitness to elite players.

Understanding the risk factors present in football referees is a crucial step towards tailoring both education and screening programs towards these match officials in order to ideally prevent episodes of sudden cardiac death or ACS while officiating a match. FIFA have introduced a mandatory yearly pre-screening for all professional players [119], though it is unclear how this is expanding to include professional referees and whether this is being adopted at the amateur level of football refereeing.

### Psychological stress faced by football referees

This review has demonstrated that both verbal and physical abuse are prevalent in football and directed towards football referees at all levels of the game. This review highlighted that the abuse received by referees is a significant reason towards them leaving the refereeing ranks. Abuse received while refereeing a match contributes to the acute psychological stress faced by football referees and as a known ACS risk factor [10,122], may increase risk of ACS events in match officials. While studies investigating emotional stressors have been highlighted above, no link has been established between this stress and documented ACS events in football referees.

### Physiological aspects to refereeing

Football referees undertake a significant cardiorespiratory load when they referee a match, covering between 9–14 km in a 90-minute period [63–74], spending a significant portion of each match at a HR > 85% of their estimated maximum HR. This has been most closely examined in the professional football referee cohort, with emerging evidence in the amateur cohort of referees. The paradox of exercise describes that cardiorespiratory effort is beneficial for long term health, but a transient increased risk of ACS events exists with acute or prolonged physical exercise [2,9]. As such, football referees may be at an increased risk of a triggered ACS events during or immediately after a match from this dual risk factor exposure. especially if chronic risk factors are also present.

### Limitations of the studies identified

#### Limitations of the review.

Studies were included in this review if they were published in English or translated to English only. Given the international nature of football, we may have excluded relevant non-English articles. There are a limited number of prospective studies identified in this review and therefore causal inference and generalisability may be limited.

#### Limitations of data.

Many of the studies reviewed were studies of professional or semi-professional football referees. While they provide useful information regarding the cardiorespiratory profile of a football referee, these officials are more likely to have dedicated time to train and receive advice from trainers, dietitians and physiotherapists etc. In contrast, amateur referees often play football and referee on the same day, or referee multiple matches in a day or across a weekend. This has the potential to impact their fitness and ability to respond appropriately in a match, while also increasing fatigue and stress on their cardiorespiratory system. There are no published studies that discuss this theme in great detail and is something that has the opportunity to be further investigated.

Secondly, a majority of the reviewed studies were of male football referees. This potentially skews the data towards this cohort of officials, though based on Australian (87%) [123] and English (92%) [124] data, a majority of referees are male. Thirdly, while cardiovascular risk factor profiles have been reported in several referee cohorts, only one study compared these with a non-referee cohort. This somewhat limits the ability to interpret this data with reference to whether football referees have increased or decreased risk compared with the broader population.

## Conclusion

The growing body of evidence highlights the cardiovascular demands, risk factors, and psychological stressors faced by football referees, although with limited data on the occurrence of cardiovascular events. Findings indicate that referees experience considerable cardiorespiratory strain during matches while also being exposed to significant psychological stress and physical abuse. Despite this, there is a notable lack of research on educational interventions aimed at promoting cardiovascular health among referees. Furthermore, referees may serve as first responders during ACS events on the field, highlighting the need for them to be well-informed about the symptoms to monitor in themselves and others, as well as to receive appropriate training for effective response.

## Supporting information

S1 Fig Preferred Reporting Items for Systematic reviews and Meta- analyses extension for Scoping reviews (PRISMA-ScR) checklist. (PDF)

S2 Fig Example search strategy (utilised in MedLine). (PDF)

S1 Table Summary table of included studies. (PDF)
