# Supplementary material for: Football (Soccer) refereeing and cardiovascular health: A scoping review
Source: PLoS One. 2026 Apr 2;21(4):e0346360. doi: 10.1371/journal.pone.0346360 (PMC13046249; doi:10.1371/journal.pone.0346360)
Supplement: S2 Fig — (PDF) [file pone.0346360.s002.pdf]

1. Heart rate/
2. Running/
3. Physical endurance/
4. Physical fitness/
5. Physical exertion/
6. Exercise/
7. athletic performance/ or cardiorespiratory fitness/ or physical endurance/ or physical fitness/
8. (heart rat* or run* or physical enduranc* or physical fitness* or physical exertion* or exercis* or athletic performanc* or cardiorespiratory fitnes* or physical enduranc* or physical fitness*).mp.
9. Death, Sudden, Cardiac/
10. chest pain/ or angina pectoris/
11. Heart arrest/
12. risk factors/ or heart disease risk factors/
13. cardiovascular diseases/ or cardiovascular abnormalities/ or heart diseases/ or vascular diseases/
14. (cardiovascular* or cardiovascular abnormalit* or heart disease* or vascular disease* or heart attack* or sudden cardiac* or heart failure* or myocardial infarction* or chest pain*).mp.
15. coronary disease/ or coronary artery disease/ or coronary stenosis/ or exp myocardial infarction/
16. (coronary* disease* or coronary stenosis*).mp.
17. stress, psychological/ or burnout, psychological/
18. Stress*.mp.
19. Anxiety/
20. Anxiet*.mp.
21. anger/ or psychological distress/ or frustration/
22. (Anger* or psychological distress* or frustrat*).mp.
23. Aggression/
24. Aggress*.mp.
25. 1 or 2 or 3 or 4 or 5 or 6 or 7 or 8 or 9 or 10 or 11 or 12 or 13 or 14 or 15 or 16 or 17 or 18 or 19 or 20 or 21 or 22 or 23 or 24
26. ((football or soccer) adj2 referee*).mp.
27. Sport official*.mp.
28. Match official*.mp.
29. 26 or 27 or 28
30. 25 and 29
