# Supplementary material for: Football (Soccer) refereeing and cardiovascular health: A scoping review
Source: PLoS One. 2026 Apr 2;21(4):e0346360. doi: 10.1371/journal.pone.0346360 (PMC13046249; doi:10.1371/journal.pone.0346360)
Supplement: S1 Table — (PDF) [file pone.0346360.s003.pdf]

Author/s	Year	Title	Journal title	Journal volume (page/s)	Context/Location	Objective	Methods	Results
A. Taylor, J. Daniel, L. Leith and J. Agnew	1988	**An investigation of the psycho-social factors preceding dropout of Ontario soccer officials**	University of Toronto. School of Physical and Health Education	-	Canada	Identifying problems that contribute to referee dropout in Ontario, Canada.	Designed a survey questionnaire for soccer referees, to examine the relationship between stress, burnout, satisfaction and their intentions to stop being officials. Completed the survey forms, in the middle and at the end of the 1987 season, to see if changes in one variable, e.g. burnout, were related to changes in another, e.g. perceived stress.	Stressful perceptions were related to levels of burnout and dissatisfaction. These in turn may lead to considering dropping out and in fact actually dropping out of refereeing.
C. Catterall, T. Reilly, G. Atkinson and A. Coldwells	1993	**Analysis of the work rates and heart-rates of association football referees**	British Journal of Sports Medicine	27 (193-196)	England	Understand work rate profile and heart rate profile of referees during a match.	Used video recordings and heart rate tracked with radio telemetry on 11 Football League matches. 14 referees included. Match recorded using a cam corder. Included sprinting, jogging, walking and moving backwards.	Distance covered in match ranged from 7977m to 10187m. Found significantly less distance covered in second half than first half. Resting heart rate pre match sitting near 100bpm. Mean heart rate during a match was 165 (+/- 8bpm). No correlation to level of the match. No difference in heart rate between halves so decrease work rate but same heart rate.
B. Hemmings and J. Graydon	1994	**Sources of stress and the incidence of burnout in national list and junior league status football referees in the 1992-93 season**	Journal of Sports Sciences	12 (195)	Canada	To examine the levels of stress and burnout present in the officiating population.	31 National list and 33 Junior league referees from Ontario took the survey. Was mailed out at pre season and midway through the season.	Found that pre season was sig lower in terms of stress of interpersonal conflicts, fear of physical harm, total officiating stress. The junior group had higher levels of time pressure stress, fitness concerns and fears of physical harms. Burnout was more prevalent in the junior group who had lower self achievement levels.
L. Johnston and L. McNaughton	1994	**The physiological requirements of soccer refereeing**	Australian Journal of Science and Medicine in Sport	26 (67-72)	Australia	Assessed the movement patterns and heart rate responses of Australian referees.	Used a grid method of tracking the referee and heart rate monitor for heart rate.	Mean distance 9408m, walking 18.9%, jogging 46.6%, running 12.1%, sprinting 6.2% and backwards 16.2%. Therefore most movement at low instenity levels. No difference in the 2 halves for total distance but sig more walking in second half. Mean heart rate was 163 then 162 bpm for the 2 halves. If using the 220-agefor max heart

								rate then spent majority of time >85% of max.
G. Ladelfa, G. Centamore, G. Platania, F. P. Maccarione, L. Carliri and M. Corsini	1994	**Referees' monitoring holter during official soccer matches**	XXVII National Congress of the Federazione-Medico-Sportiva-Italiana	-	Italy	To examine the strain the heart faces while refereeing	8 referees wore Holter monitor during a match.	Found resting heart rate 87bpm pre match and 94bpm post match. Average heart rate first half was 165 and second half 167. Heart rate during admonishment was 160, same with expulsions, 170 when giving a penalty and 167 when a goal scored.
S. D'Ottavio and C. Castagna	2001	**Physiological load imposed on elite soccer referees during actual match play**	Journal of Sports Medicine and Physical Fitness	41 (27-32)	Italy	Examine the activity profile and physiological responses of top Italian competitions.	18 top level Italian referees. Mean age 37.5. Used 2 cameras to track movements during the 1991-92 season.	Looked at standing, walking, jogging, medium intensity run (13.1-18km/h), high intensity run (>18km/h), maximal speed >24km/h, backwards movement. Mean heart rate 163bpm, correlated to 89.1% of HR max. No sig differences between the halves for HR. Sig differences seen in 0-15 vs 15-30 and 0-15 vs 30-45min segments of the match. Not seen in 0-15 vs any second half segment. Stood still for 968 seconds (16.9% of the match). Mean distance covered was 11376m. Walked forward 889m. Maximal speed runs 608m.HIA (anything above 13.1km/h) 4749m or 41% of total distance. Difference in distance covered in each half not significant.
S. D'Ottavio and C. Castagna	2001	**Analysis of Match Activities in Elite Soccer Referees during Actual Match Play**	Journal of Strength and Conditioning Research	15 (167-172)	Italy	Examine in detail the activity profile of top-level Italian soccer referees during first division (Series A) official matches	33 top level Italian referees. Mean age 37.8 and observed 1-6 times on a match.	14.6% of match time stood still. Mean distance 11469m. Decrease in distance covered per half of 4.1% was significant. Ran at maximal speed for 202m in first half and 225m in second half. 1973m at HIA or 17.2% of total distance covered. Decrease in distance covered in final 15 mins of both halves.

P. Krustrup and J. Bangsbo	2001	**Physiological demands of top-class soccer refereeing in relation to physical capacity: effect of intense intermittent exercise training**	Journal of Sports Sciences	19 (881-891)	Denmark	To examine the physical demands and physiology of top class referees.	27 referees over 43 matches in top 2 Danish leagues using heart rate monitor, GPS. Did testing pre and post training and compared it to the matches.	Total distance 10007m, 1.67km was HIA. HIA and backwards decreased in second half sig. Mean heart rate 162 bpm, which was 85% of max heart rate. Amount of HIA was related to level of Yo-Yo testing achieved. After intermittent training, total distance and HIA was greater in match, mean heart rate was lower.
P. Folkesson, C. Nyberg, T. Archer and T. Norlander	2002	**Soccer Referees' Experience of Threat and Aggression: Effects of Age, Experience, and Life Orientation on Outcome of Coping Strategy**	Aggressive Behavior	28 (317-327)	Norway	To understand the threat and aggression that local referees face while refereeing a match.	107 local referees recruited. Undertook a survey to answer questions about what causes them to feel threatened at a match and to elaborate on their previous experience, and life outlook.	Three main threats were ID'ed: players, coaches and spectators. Main way of threatening was verbal threat and threat of physical abuse. Noted to impact pre match focus, concentration and confidence. This was offset by the age, experience and life orientation of the referee. Younger referees were more prone to the impacts of the abuse and those with more pessimistic outlook tended to have worse performance.
C. Castagna and G. Abt	2003	**Intermatch variation of match activity in elite Italian soccer referees**	Journal of Strength and Conditioning Research	17 (388-392)	Italy	To examine whether elite-level referees experience significant intermatch intensity-related variation in physical performance, assumed as distance covered.	14 referees observed on minimum 3 and max 6 Serie A matches (total 65 matches). Recording cameras tracked referee movements and broke down into different types of movement.	Referees can reach 67-88% of maximal oxygen intake and HR. Found elite Italian referees have 17% of their activities in their peak energy expenditure. Conflicting data on changes in parameters in first vs second half, some see decrease in distance covered, HR etc. No change in high intensity activity across matches. Points towards referees altering their technique for movements based on the match without impacting ability to complete HIAs. Looks to be sparing activities, as in saving themselves for what they need onwards.

C. Castagna, G. Abt and S. D'Ottavio	2004	**Activity profile of International-level soccer referees during competitive matches**	Journal of Strength & Conditioning Research	18 (486-490)	International	Examine the physical load imposed on International referees during a competition.	13 FIFA referees from across Europe age 38 with >5yrs at the top level involved. Also got 13 National referees refereeing in Serie A. Referees observed by 2 cameras as above and tracked movements.	National referees stood still longer and used lower intensity than International referees. Medium intensity coverage dropped sig in the second half (1829 to 1684m), but HIA and sprints showed no difference in half to half analysis. Appeared overall to show international matches to be less demanding than national matches. Note the wide range of distance covered in the literature. Harley found 7.6km in English county referees, EPL found 9.5km, Johnston and McNaughton for Aus found 9.4km.
W. Helsen and J. Bultynck	2004	**Physical and perceptual-cognitive demands of top-class refereeing in association football**	Journal of Sports Sciences	22 (179-189)	Europe	"The specific aims of this study were to: (1) examine the physical load imposed on both referees and assistant referees during the matches of the Euro 2000 Championship; and (2) assess the perceptual-cognitive demands of top-class refereeing during all the 31 matches of the Euro 2000 Championship, expressed as the number and type of visible decisions."	17 UEFA referees and 17 UEFA Assistant Referees included in the study. Heart rate measurements taken during the match. All decisions in a match were tracked by video taping the match. Referees mean age 40.2, while AR's 41.3. BMI 24.2 vs 24 respectively.	Can be difficult to compare distance covered data as more recent studied have more activity groups than studies of the 80's and 90's. Players in EPL cover avg 1.5km more than South American players so this also impacts the distance referees have to cover. Sig difference for heart rate at rest, referees lower than Assistant Referees. Referees were 85% of maximal heart rate and AR's 77%. Workload for referees increased towards the end of each half. Average 137 decisions per match. Referee by themselves had to make 44.4 decisions, with throw ins being the highest total decision overall. All decisions, 64% involved communication with AR or 4thy. HR max is higher during the match than any training or lab session can recreate. 220-age for max HR is good approximate but not always perfect when compared with a test of HR in a lab setting.

C. Castagna, G. Abt, S. D'Ottavio and M. Weston	2005	**Age-related effects on fitness performance in elite-level soccer referees**	Journal of Strength & Conditioning Research	19 (785-790)	Italy	To investigate the possible age-related variations in fitness performance in a group of highly competitive soccer referees to obtain information to be used to guide selection and training prescription.	36 elite Italian referees studied, the entire group able to referee Serie A and B that year. Grouped into 31-35, 36-39 and 40-45 yo. Male referees. 37 +/-3 years. All were part time and trained at least 3 times a week. All completed fitness tests as set out by UEFA. 50m and 200m sprints and 12 minute run.	85-95% of maximal heart rate range covered from 9-14km. Paradox of being at their best when players have often retired from the game. Avg age of referees at 2002 World Cup from Quarter Final onwards was 41.4 +/- 4 years. Endurance test no differences in groups. Marginally quicker for young group over the 50m sprint. 12minute run test showed negative correlation with age. The older referees developed a well developed submaximal aerobic performance.
J. Mallo, J. M. Garcia-Aranda and E. Navarro	2006	**Analysis of the physical demands placed on football referees during competitive matches**	European Journal of Human Movement	17 (25-39)	International	To examine the behaviour of several kinematical and physiological parameters throughout competitive matches in football referee	Video fo 12 games from Under 17 WC in 2003. Heart rate recorded at 5s intervals.	Found impairment of activity in 3 sections; after the most intense 5min period of the match, in first 15 mins of second half the distance covered, HIAs and mean HR were sig down and second half saw global reduction of intensity based on HR and HIA completed.
S. Müniroglu	2007	**The Relation Between Heart Rate and Running Distances of Football Referees During the Matches**	International Journal of Applied Sports Sciences	19 (7-15)	Turkey	The relationship between distance, heart rate and anaerobic threshold in amateur referees.	10 local referees, mean age 27.03. tracked heart rate with heart rate monitor, distances tracked on replica field with markings.	Found increase in distance in second half compared to the first half.
G. E. Fernández Vargas, A. Inácio da Silva and M. Arruda	2008	**Anthropometric profile and physical fitness of the professional referees Chilean soccer**	International Journal of Morphology	26 (897-904)	Chile	To analyse the anthropometry and physical fitness of Chilean first division referees.	11 male referees. Mean age 34.54, BMI 25.14. Body fat percentage 15.44%.	The referees held typical body shapes and were able to complete the FIFA fitness testing. Their body fat percentage may limit their endurance capacity but are able to function at normal levels.
G. Galanti, A. Pizzi, M. Lucarelli, L. Stefani, M. Gianassi, V. Di Tante, et al.	2008	**The cardiovascular profile of soccer referees: an echocardiographic study**	Cardiovascular Ultrasound	6 (1-5)	Italy	To evaluate the morphological and functional profiles of professional football referees.	120 professional Italian referees. Compared to 120 players.	LV mass index and RV dimensions were significantly greater in referees. Referees were taller than players. When comparing Div 1 referees and players only, LVMI, LA dimension and aortic root were sig greater in players. A referees heart can be considered physiologically an athletes heart. See CV adaptations to keep up with the players.

P. Gardener	2008	**RESPECT Campaigns in Football**	World Soccer	16	England	-	-	Discussing the Respect campaign in EPL referees from 2008. Raises the issue that it's hard to combine the respect for officials when it needs to come from the people that are able to have their concerns regarding officials conduct.
S. Gencay	2009	**Magnitude of psychological stress reported by soccer referees**	Social Behavior & Personality: an international journal	37 (865-868)	Turkey	To understand the magnitude of stress faced by referees and Assistant Referees.	156 referees from the Turkish FA responded to an online survey.	Perceived stress ranged from very little to a moderate amount. No differences between referees and Assistant Referees in perceptions of stress. Didn't elaborate on the causes of stress nor on the experience of referees or the competitions that they officiate in.
P. Krustrup, W. Helsen, M. Randers, J. Christensen, C. MacDonald, A. Rebelo, et al.	2009	**Activity profile and physical demands of football referees and assistant referees in international games**	Journal of Sports Sciences	27 (1167-1176)	International	To examine the physiology and exercise levels of referees in an international match and see if distance related to closeness to the ball.	15 referees and 15 Assistant Referees. Used time motion analysis and took physiological markers.	Total distance 10270m, with 1.92km of HIA. AR's 6.76km and 0.97km HIA. Sprinting distance not different between the 2. mean HR higher for referees than AR's, 150bpm vs 123bpm. Backwards and sideways movement decreased in final 15mins but not HIA.
J. Mallo, E. Navarro, J. M. G. Aranda and W. Helsen	2009	**Physical Demands of Top-class Soccer Assistant Refereeing during High-standard Matches**	International Journal of Sports Medicine	30 (331-336)	International	To examine the kinematical and heart rate response of top-level assistant referees during an international high-standard senior FIFA tournament. An additional purpose was to investigate the effect of the level of the competition and the displacements of the ball on the kinematical performance of the assistant referees.	18 top Assistant Referees (AR) mean age 38.67, all FIFA AR's and avg 5 years of international experience. Assessed during Confederations Cup in Germany 2005. Matches videoed to track movements and heart rate recorded at 5s intervals.	Assistant Referees should be considered a different population based on their movements and distance covered. Other studies have found that they tend to cover 6-7km with 16-20% at HIA, with mean heart rate 137-140bpm. In this study mean distance was 5752m. Covered sig more distance in first half than second half. On average stood still for 48% of match, walked for 25% of match, jogged for 16.6% cruised for 6.1% and high speed for 4.4%. Sideways for 29.7% of match. No difference in distance covered by the ball in each half. Mean HR 140bpm which was 78% of HR max, decreased from 143 to 137 in second half which was sig. 20% of match >85% HR max. Sideways was for 1.7km and is shown to be more demanding than forward movement. Showed decrease in

								distance and HR in second half but not in HIA or sideways movement which implies a working out of how to move best.
J. Mallo, E. Navarro, J. M. G. Aranda and W. F. Helsen	2009	**Activity profile of top-class association football referees in relation to fitness-test performance and match standard**	Journal of Sports Sciences	27 (9-17)	International	To perform match analysis and compare activity profiles of the elite-level soccer players and referees, analyze foul plays per player position and pitch location, and foul decision-making factors during nationally held professional league tournament in Korea.	Cameras tracked matches at Korean national competitions. Data from 14 referees mean age 37.5.	Currently see about 60 mins of actual play time in football. Referees covered 10.5km and players 11.9km. More slow walking by referees and sprinting and jogging by the players. Distances covered in 15 min periods from 30min onwards were seen in both players and referees. Similar % in each of the movement zones other than those listed above.
M. Voight	2009	**Sources of stress and coping strategies of US soccer officials**	Stress & Health: Journal of the International Society for the Investigation of Stress	25 (91-101)	USA	Attempt to understand how sports officials cope with stress and abuse.	Questionnaires to over 200 officials.	Top stressors reported were balancing refereeing with family, making a controversial call, conflict between refereeing and work. Steps to cope included thinking hard about what to do and asking other officials. Also discussed relaxation, time management, restructuring unproductive thoughts.
E. Bambaeichi, A. R. Movahedi and M. Abedini	2010	**The relationship between cardiovascular risk factors and trait anxiety of Iranian referees and assistant referees in Premier League Soccer**	British Journal of Sports Medicine	44 (22)	Iran	To explore relationship between trait anxiety and cardiovascular risk factors	RISKO questionnaire and also Zung Self-rating Anxiety scale.	25 referees and 43 Assistant Referees, mean age 36.7, no difference in trait anxiety score between referees and Assistant referees.

M. Weston, C. Castagna, F. M. Impellizzeri, E. Rampinini and S. Breivik	2010	**Ageing and physical match performance in English Premier League soccer referees**	Journal of Science & Medicine in Sport	13 (96-100)	England	"Aims to 1) examine the effect of age upon the physical match performances of soccer referees in relation to the distance from fouls and the ball over the course of four consecutive seasons; and 2) examine the effect of age upon the physiological load imposed upon soccer referees during competitive matches."	22 EPL referees, 778 match observations. Used ratings of perceived exertion, heart rate, to give a mean score for the match. Used a semi automated video tracking system for the movements. Looked at total distance, high intensity >19.8km/hr, sprint count >25.2km/hr, top sprint speed, distance from ball. Recorded RPE using Borg's 10 point scale.	At time of paper, age related retirements in force for referees with no specific scientific backing. Negative correlation between age and total distance, high intensity running, and sprint speeds. The younger referees covered more distance than the other 2 groups. Older referees sig slower in top speed than other 2 groups. Match intensities were the same across the board. Therefore see an age related decline in refereeing physical ability. However the older referees were still close to the ball, highlighting knowledge of how to use movements to stay close to play and preserve energy.
Y. Can, Ü. ÇOban and F. Soyer	2011	**A research to find out effects of negative emotionality on stress of referees working in Turkey**	Nigde University Journal of Physical Education & Sport Sciences	5 (165-174)	Turkey	To examine the negative emotionality and stress of referees in Turkey	104 referees (86 male referees), 25 question survey.	Relationship between work stress and negative emotionality.
V. Di Salvo, M. R. Carmont and N. Maffulli	2011	**Football officials activities during matches: A comparison of activity of referees and linesmen in European, Premiership and championship matches**	Muscles, Ligaments and Tendons Journal	1 (106-111)	England	"Compare the activity of referees and assistant referees officiating matches in three different competitions: UEFA and Champions League European games, the English Premiership and the English Championship during the 2005/2006 football season, to determine whether there was a difference in the activity of officials between the different competitions."	Notational analysis using 8 video cameras around the ground. HIA was >19.8km/h.	Analysed 328 matches, 68 referees and 170 Assistant Referees. Shorter distances covered in the second half and sig less jogging (7.3-14.4), running (14.5-19.8) and high speed running (19.9-25.2) in the second half but no difference in sprinting (>25.3). Found English Championship referees covered more distance than EPL or European Comp matches. Showed 7.3% of activity was greater than 19.9km/h.
L. Lategan	2011	**Physiological profiles of South African soccer referees and assistant referees**	African Journal for Physical, Health Education, Recreation & Dance	17 (675-693)	South Africa	To understand the physiology of South African football referees and understand the level of activity they're required to input.	7 referees and 13 Assistant Referees on the National panel included.	They had expected levels of VO2 Max and heart rate max and heart rate mean as per the previous literature, they were of expected flexibility and had approximately normal levels of activity breakdown per activity.

J. A. Ruiz Caballero, E. Brito Ojeda, J. M. Garcia-Aranda, J. Mallo, W. Helsen, S. Sarmiento, et al.	2011	**Echocardiographic study of structure and functional cardiac profile of football referees**	Journal of Sports Medicine and Physical Fitness	51 (633-638)	Spain	Describe and analyse through echocardiography the structure and function of hearts of referees from Las Palmas.	54 national level referees. All with at least 5 years at senior levels. Mean age 28.5,BMI 24.9,	Herat rate at rest was 58.55bpm, average heart rate lower than that reported in Italian referees. Diameter of ventricular chambers bigger in referees than in players. May be due to difference in training workload etc. All findings were within the normal ranges expected.
J. Barbero-Alvarez, D. A. Boullosa, F. Y. Nakamura, G. Andrin and C. Castagna	2012	**Physical and physiological demands of field and assistant soccer referees during America's cup**	Journal of Strength & Conditioning Research	126 (1383-1388)	Venezuela	Examine physical and physiological demands of referees and Assistant Referees.	GPS and heart rate monitor on referees and Assistant Referees, avg age of referees 40y.	Approx 10% at high intensity and cover 11-12km, referee mean 10.197km, Assistant Referee 5.819km, found no difference between halves for the activities, couldn't differentiate between types of activities , see correlation between heart rate level and type of activity.
M Bizzini, C Schmeid, A Junge and J Dvorak	2012	**Precompetition medical assessment of referees and assistant referees selected for the 2010 FIFA World Cup**	British Journal of Sports Medicine	46 (5) 374-374	Switzerland	Assess pre-competition medical assessment utility in referees attending the 2010 World Cup.	30 referees and 60 assistant referees underwent testing, following FIFA standardised protocol over 1 month period in 2010. 90 male referees, average age 39.1.	One referee had T1DM, and one had HTN. 10 referees reported Fhx of CHD, 3 reported cardiomyopathy, 5 for stroke, 18 for HTN and 18 for diabetes. On exam, 2 had "suspicious" murmurs, 20 had at least one lab findings of lipids (2), LFTs (9), morphologically suspicious RBCs (2), decreased plt (1), anaemia (2), elevated urea/uric acid (3) or increased CRP (1). 32 showed at least one path finding on exam 4 with Brugada like pattern, some with TWI in lateral leads. 5 had hypertrophic LV with impairment of diastole, 2 had no impairment of diastole. Others included dilation of aortic root (2), RV hypertrabecoluation (2), MVP (1), All had excellent physical capacity. No exercise induced ECG changes.
D. Boullosa, L. Abreu, J. Tuimil and A. Leicht	2012	**Impact of a soccer match on the cardiac autonomic control of referees**	European Journal of Applied Physiology	112 (2233-2242)	Spain	To examine the fitness levels and cardiovascular parameters of football referees from Spain.	16 sub-elite referees from Spanish 3rd division, with min 6 yrs experience and trained 0-2 days a week.	Those with greater physical fitness based on VO2 max have greater heart rate variability recovery, heart rate sig higher in match day than rest day, majority of match time at >75% HRmax, with females spending 86% time there. Also less heart rate variability prior to and following a match than on rest day.

								Those with increased variability on rest day had increased response to stressful stimuli during the match.
F. Halabchi, R. Mazaheri, M. A. Mansournia and T. S. Barghi	2012	**Study of selected health and performance related parameters among football referees of Iranian premier League**	Pejouhandeh	17 (1-7)	Iran	To assess the selected parameters of fitness as well as risk factors of musculoskeletal problems among all referees of Iran's Premier League during season	Cross-sectional study. 78 participants, 32 referees, rest AR's. Mean age 37, BMI 23.6,	840,000 registered referees worldwide. Observed muscle tightness in 38 participants. Single leg balance test abnormal in 56 cases.
R. Adnan, N. Muzayin and N. Sulaiman	2013	**Analysis of Movement Pattern among Referee in 2012 Malaysian Cup**	Journal of Human Sport & Exercise	8 (642-650)	Malaysia	Analyse whether movement type related to total distance covered by referees.	7 matches from Malaysian Cup 2012. Used computer system to track distances covered and types of movement made.	137 decisions a match (avg 3-4 per min). 47% running, 42% walking, 8% backwards, 3% peak
E. C. Costa, C. M. Vieira, A. Moreira, C. Ugrinowitsch, C. Castagna and M. S. Aoki	2013	**Monitoring external and internal loads of brazilian soccer referees during official matches**	Journal of Sports Science & Medicine	12 (559-564)	Brazil	To assess the external and internal loads of Brazilian soccer referees during professional official matches.	11 Brazilian soccer referees, age 36.2 with 8.3 years of experience.	Physical demand - external load. Internal load - heart rate and rating of perceived exertion (RPE). Only found a significant difference in average speed between first and second half, not in total distance nor maximum speed. During 95% of the match, the referees were at >80% of heart rate max. Average distance covered was 10.5km. Mean speed 6.5km/hr and max speed 19.3km/h. Time spent at 90-100% of heart rate max was higher in the first half than the second half. Mean heart rate 165bpm. Session RPE varied from 5.5 to 8 so hard to very hard.
B. T. Johansen and T. Haugen	2013	**Anxiety level and decision-making among Norwegian top-class soccer referees**	International Journal of Sport and Exercise Psychology	11 (215-226)	Norway	To understand the level of anxiety in Norwegian top class referees and see if it could be predicted based on experience or level of officiating.	83 referees, 73 male. Mean age 33.2 completed an online survey. Used STAI for anxiety.	Mean level of anxiety for the respondents was 28. Referees from the top level scored higher (worse anxiety) than those in the second level. Those that perceived themselves to be worse than their colleagues score sig worse on anxiety. Most reported that the noise of the crowd, failure or mistakes and aggressive players did not change their refereeing.

R. Neil, P. Bayston, S. Hanton and K. Wilson	2013	**The influence of stress and emotions on association football referees' decision-making**	Sport & Exercise Psychology Review	9 (22-41)	England	"To explore the stress and emotional experiences of football referees, informed by Lazarus' (1999) CMR Theory of Emotions. Specifically, to examine the influence of stress and emotions on decision-making through identifying the stressors encountered, the consequent appraisals, emotions felt, and coping strategies used."	1 female and 3 male referees in different levels in the UK. 2 amateur and 2 professional, age and experience withheld. Interviews held with participants probing the key areas discussed in the results section.	Crowd factors influencing the decisions included spectator abuse and collective crowd noise. All 4 said they were prone to worse emotions if receiving direct verbal abuse from spectators. The amateur referees showed signs of retribution and counter attacking to the abuse by way of decision making. The higher qualified referees used emotion centred techniques to cope. Lack of confidence lowers perception to cope with difficult situations. Decisions were more likely to go against the team where a player or official had just confronted the referee during the match. Professionals used problem focussed coping strategies.
B. Blumenstein and I. Orbach	2014	**Development of Psychological Preparation Program for Football Referees: Pilot Study**	Sport Science Review	23 (113-125)	Israel	Israeli Football Association wanted a psychological preparation program for their referees - aiming to create one.	10 referees mean age 28.8 with mean experience of 8.7y involved, had 3 parts - educational, acquisition and evaluation.	Most stressful thing was fear of making mistake 70%, anxiety from fans and assessor 60%, low self confidence 40%, 80% said worst time was first 10-15 mins of a game, also had fear of a bad report from their assessor, found higher self confidence in ability to cope with stress.

L. A. Slack, J. Butt, I. W. Maynard and P. Olusoga	2014	**Understanding mental toughness in elite football officiating: Perceptions of English Premier League referees**	Sport & Exercise Psychology Review	10 (4-24)	England	"The purpose of the study was to: (a) identify situations perceived by elite football referees to require MT, and (b) document the 10 associated behaviours and cognitions utilised by referees within these situations."	15 EPL referees, mean age 42, interviewed face to face or online. 3 were retired within last 2 years.	Superior set of behaviours and cognitions in sports participants with mental toughness in sport. Previous work in referees highlighted key characteristics: coping with pressure, achievement striving, resilience, high work ethic, robust self belief, tough attitude and sport intelligence. Found 3 key areas needing mental toughness: pre match situations, during match and then post match situations. Pre match included meeting captains etc, having a high intensity match. During the match it was about verbal and non verbal actions with those involved in the game, also critical incidents/flash points. Post match situations involved media criticism or players criticism. There were also off field situations needing toughness, scheduling, programming, training, pressures form the Select Group, Also a range of general life things to consider family, sacrifices etc. The mental toughness behaviours included being cool and calm, explaining expectations to teams, strong body language. Mental toughness cognitions involved, thriving in front a big crowd, focus on goals while refereeing, grounding from other parts of life to say it's just a game etc. The pre match area seemed to be a big area of focus for the referees in terms of where their mental toughness is used. Also discussed blocking out crowd noise or focussing in blocks of 5 mins.
J. Yanci-Irigoyen	2014	**Changes in the physical fitness of soccer referees: a longitudinal study**	International Journal of Sports Sciences	10 (336-345)	Spain	To understand the evolution of fitness in football referees by cardiovascular capacity, jumping ability and acceleration capacity.	16 referees included in the study. Mean age 28.37, from different levels of Spanish football.	No changes in VO2 max or time to exhaustion over the 3 seasons. Ability to accelerate 5m and 15m was sig worse over the 3 seasons. Loss of muscle stretch in lower extremities too.

L. Lela Maskhulia, V. Akhalkatsi, K. Chelidze, Z. Kakhabrishvili, M. Matiashvili, N. Chabashvili, et al.	2015	**Pre-participation cardiovascular screening of football referees: Clinical findings and experience**	European Journal of Preventive Cardiology	1 (S43)	Georgia	To analyze cardiovascular findings obtained in the pre-participation screening (PPS) of the national category Georgian football referees.	67 professional Georgian football referees. Pre participation screening with history, exam, ECG and TTE. Mean age 37.2, BMI 24.7.	Hypertension at rest in 7.5%. T-Wave inversion in 3% of participants during exercise. MVP in 6%, nil complained of symptoms related to myocardial ischaemia. Exercise hypertension in 7 referees.
T. Parsons and A. Bairner	2015	**You want the buzz of having done well in a game that wasn't easy: a sociological examination of the job commitment of English football referees.**	Movement & Sport Sciences	87 (41-52)	England	To understand the driving factors behind football referee retention and departure rates in English football.	12 referees from all levels of English football. 22-61yo, from EPL to amateur.	Football Association (UK) keeps referees 14-19 but struggles with the early to mid 20's referees. Formation of the FA in 1863, 1866 the role of the referee became more obvious and formal. 2001 was the first year English referees went professional. Want respect, pay and involvement from peoples involved to keep going. A mentor is valuable too. Referees have stayed in the game, based on autobiographies, due to the social aspect, being involved with the game and the thrill of the contest. From the interviews, the main result for staying involved was enjoyment of what they were doing. Fits with other data who found 90% stay involved fdue to enjoyment. Higher level referees seemed to pay more attention to their mistakes than those at the grassroots. 2 referees indicated they wanted to give the game away and this was due to no enjoyment anymore. They also stated personal factors like job and age as reasons for wanting to step away. Still too much abuse at the grassroots and FA not clamping down on it. Lack of backing by the FA noted at all levels. Between 2007 and 2010 there has been a 579% decrease in referees aged 20-24. Those that want to leave describe a means to an end for refereeing. All referees said they enjoyed the game when it was challenging and were bored otherwise. Lots of aspiration to move up the ranks and often being

								stuck at a level for a few years was enough to become frustrated and want to leave. Only 1 referee put the emphasis on the pay as a reason for doing this. Social supports highlighted by all referees as crucial to what they do.
I. Pedrosa and E. Garcia-Cueto	2015	**Psychological aspects in elite referees: Does the wage affect their emotional well-being?**	Sports Psychology Magazine	24 (241-248)	Spain	Compare the psychological and wellbeing levels of referees across sports based on their sporting income.	300 referees, 41% soccer, 36% swimming, 23% canoeing in Spain. 73% male.	Women demonstrated more symptoms to burnout type disorders, had lower levels of perceived social support. Soccer referees had sig better levels of self worth, social support, burnout compared to the other sports.
L. A. Slack, I. W. Maynard, J. Butt and P. Olusoga	2015	**An Evaluation of a Mental Toughness Education and Training Program for Early-Career English Football League Referees**	Sport Psychologist	29 (237-257)	England	To examine the effectiveness of a long-term MTETP intervention tailored for early-career, level one EFL referees.	3 EFL referees and their coach. Looked at mental toughness, sports-focussed mental toughness and reports from assessors to see impacts.	Higher mental toughness after intervention than before. All 3 reported improvements in referee specific mental toughness. Higher behaviour ratings. Increasing the level of self reflection was important to the role too.

D. Castillo, J. Camara, J. Castellano and J. Yanci	2016	**Football match officials do not attain maximal sprinting speed during matches**	Kinesiology	48 (207-212)	Spain	"The objectives of this study were, on the one hand, to analyse the differences between the maximum movement speeds reached by referees both in matches and in a sprint test, and on the other hand, to determine the differences in performance between field referees and assistant referees in 20-metre and 30-metre sprint tests."	20 referees from Spanish third division. Age 28.4, BMI 23.51, 12 referees and 8 AR's. Completed 30m straight line sprint test in pre-season. Then also used GPS monitoring to track speeds during matches in season.	State again average 11070+/-808m per match with 889m in high intensity region (>19.8km/h). And perform 21-30 sprints per match. Sprints rarely last longer than 4 seconds. Implication that testing over the 40 or 50m sprints is not representative of real match situations. Total group sprint speed in the test was 29.45km/hr, no differences seen in time to complete sprints between the two groups. Both groups were quicker in sprint testing than in match. Referees top speeds in the test was 26.34km/hr, with a difference of means to in match of 3.3 km/hr. Assistant Referees saw difference of means of 4.99km/hr.
D. Castillo, J. Yanci, J. Camara and M. Weston	2016	**The influence of soccer match play on physiological and physical performance measures in soccer referees and assistant referees**	Journal of Sports Sciences	34 (557-563)	Spain	Assess the impact of soccer refereeing on physical and physiological parameters.	Used GPS tracking, blood lactate, tympanic temperature, sprint speeds and vertical jump. Referees from Spanish third division involved over 8 of their matches.	Saw increase in post match blood lactate levels, longer 15m and 30m sprint times and non dominant leg jump height.
R. Mazaheri, F. Halabchi, T. S. Barghi and M. A. Mansournia	2016	**Cardiorespiratory Fitness and Body Composition of Soccer Referees; Do These Correlate With Proper Performance?**	Asian Journal of Sports Medicine	7 (1-5)	Iran	To assess the cardiorespiratory fitness and body composition and the potential correlation between these parameters and performance scores of all referees of Iranian Premier Soccer league season 2009-2010	78 male Iranian referees, 32 centre referees, and 46 AR's. Avg age 36.96. Mean BMI 23.64. Compared their parameters to their scores as allocated by match assessors.	In each week of play, more than 1 million referees take to the field. With more than 840,000 registered. Run similar distances to a midfielder. 29.5% were overweight and none obese. Looked at respiratory markers like VO2 max and FVC etc. Found the fitness status of the top level referees is high enough to cover the match and not deteriorate despite increased age. Found no sig links between any body parameter and their overall refereeing score/physical score.
I. Pedrosa and E. Garcia-Cueto	2016	**Burnout Syndrome in Elite Referees: The Spanish Professional Soccer League (LFP) under Analysis**	Ibero-American Journal of Diagnosis and Evaluation - Psychology and Assessment	2 (59-68)	Spain	Understand the level of burnout in Spanish professional referees and the link between burnout and social supports.	123 (all the professional) referees from Spain undertook burnout questionnaire.	Prevalence of burnout of 2.44%. No link between amount of experience or age or length of time refereeing and burnout. Head referees had more tendencies towards burnout.

M. Rebolé, D. Castillo, J. C. Cámara and J. Yanci	2016	**Relationship between the cardiovascular capacity and repeated sprints ability in high-standard soccer referees**	Ibero-American Journal of Physical Activity and Sports Sciences	5 (49-64)	Portugal	Compare he performance in lab testing and a repeated sprint ability test of referees.	12 high division referees, mean age 28.8, BMI 22.82.	Obtained 59.5 ml/kg/min VO2 max. Found positive correlation between cardiovascular variables and RSA times. Decreased ventilatory threshold correlated negatively with fatigue index.
T. K. Bozdogan, A. Kizilet and B. Bicer	2017	**The effect of morphological characteristics on the physical and physiological performance of Turkish soccer referees and assistant referees**	ERPA International Congresses on Education (ERPA) Budapest	-	Turkey	To discern whether anthropometry makes a difference to physical performance	Mean age 31.8, from Turkish leagues , Assistant Referee mean age 37.4, underwent physical testing such as Yo-Yo testing.	Elite referee runs 110 high intensity moves a match for <2s each on average, 158 male referees and 55 male Assistant Referees, avg BMI 23.5, both body fat and BMI negatively correlated with RSA scores, ideal BF % is <12%, with some papers findings up to 20% in Greek referees.
B. Dolański, A. Szwarc, B. Heinig and M. Sitek	2017	**Physical activity profile of the referee and the assistant referee during official football matches**	Baltic Journal of Health & Physical Activity	9 (97-105)	Poland	To define motor activity profiles of the main referee and the assistant referee during official championship matches organized by European football federations.	Activity of 10 referees and 10 Assistant Referees was observed in 2016 period. Age range 26-30. Level of experience 7-10 years. Assistant Referees were 25-28 yo and experience 5-7 years. Tracked using a heart rate monitor and GPS.	2009 Confederations Cup found increase in incorrect decisions in second half 9.3 to 17% and most errors in final 15 mins 23%. The referee covered 7.75 km and heart rate avg 162bpm while the Assistant Referees avg 4.40km and 137bpm. Referees had a higher mean speed and that Assistant Referees had a higher fastest speed avg than the referees.
L. Gianturco, B. Bodini, V. Gianturco, G. Lippo, A. Solbiati and M. Turiel	2017	**Left ventricular longitudinal strain in soccer referees**	Oncotarget	8 (39766-39773)	Italy	To define the characteristics of LV myocardial mechanics as assessed by 2D STE in a group of top level referees and to correlate those findings with a traditional marker of aerobic power such as VO2max.	20 referees from Italian federation. Mean age 33.87, BMI 22.4, nil history of heart disease. Had an ECG and echo.	Prolonged exercise changes the heart by increasing mass, wall thickness and diameters of chambers. Referees had normal values of diameters, volumes and cardiac mass.

V. Gouttebarge, U. Johnson, P. Rochcongar, P. Rosier and G. Kerkhoffs	2017	**Symptoms of common mental disorders among professional football referees: a one-season prospective study across Europe**	Physician and Sportsmedicine	45 (11-16)	Europe	"To determine the prevalence and one-season incidence of symptoms of Common Mental Disorders (distress, anxiety/depression, sleep disturbance, eating disorders, adverse alcohol use) among European professional football referees. A secondary aim was to explore the view of European professional football referees on consequences, support, and needs related to these symptoms of CMD."	Observational cohort study with data sampling points over one season. Professional football referees from a number of European countries. Distress by distress screener. GHQ-12 used for anxiety/depression. PROMIS for sleep disturbance. Eating disorders screen for primary care. Used AUDIT-C for alcohol intake. Ended with 292 who completed the whole thing but initially 391 started the study. Mean age 33. Avg 7 years in professional football.	Baseline stats for distress 6%, anxiety/depression 12%, sleep disturbance 9%, 19% eating disorders and 17% for averse alcohol use. During the follow up period distress to 10%, anx/dpe 16%, sleep disturbance 14%, eating disorders 29%, 8% averse alcohol use. More than 90% thought poor mental status would impact decision making but only 18% did seek help for mental disturbances. rates are similar to studies completed in football players and rugby union players (both retired or still plying). Symptoms of distress and common mental disorders all parallel studies of general populations in other areas.
D. Niederseer, A. C. Franz, D. Keller, A. Junge, M. Bizzini, J. Dvorak, et al.	2017	**Cardiovascular screening of football referees for the 2014 FIFA Football World Cup in Brazil - Time for revision of current screening concepts in competitive master athletes?**	European Journal of Preventive Cardiology	24 (S48)	International	To assess elite referees for detection of potential cardiac disorders.	Examined all 156 preselected referees for the 2014 World Cup. Did history, exam, ECG, blood tests, echo and exercise ECG. All were male, average age 37.4. BMI 23.4.	43 had abnormal lab findings, 25 had dyslipidaemia, 17 had impaired fasting glucose and 1 had new diagnosis T2DM. 7 had BP >140/90. 44 had pathological ECG findings, 16 had T-Wave inversion, 12 had ST segment depression, 1 had pathological Q waves. 4 had LAD. On echocardiogram, 6 had dilated aortic roots, 1 had a RCA from L sinus with intramural route. None had exercise testing abnormalities.
Y. Şirin and E. Dosyilmaz	2017	**Investigation of job satisfaction and burnout levels of Turkish Super League football referees**	Nigde University Journal of Physical Education & Sport Sciences	11 (87-96)	Turkey	To examine burnout and job satisfaction, which are factors that may influence football referees' decisions, in terms of some variables	80 upper division Turkish referees. Burnout scale abbreviated used. SFMS used for job satisfaction. Most were 36-41yo, avg age 38.8. Most had 10+ years of refereeing experience.	Burnout first described in 1981 and can manifest in physical symptoms or in office burnout with anxiety depression etc. See decreased job satisfaction etc. Job satisfaction in influenced by many things. Married referees had higher intrinsic satisfaction than unmarried. Single referees had sig higher burnout levels than married referees. Centre referees had higher burnout than AR's. No difference in occupation, age. Sig difference in refereeing experience for burnout. Generally had high satisfaction

								levels though. Marital status follows from previous studies due to it showing a more consistent life etc. Sig difference between those who had experience as a player and those that didn't. Believe that because they have played it they get more satisfaction and know the game better.
G. Soriano, Y. Ramis, M. Torregrosa and J. Cruz	2017	**Support Against Stress: How We Can Help Referees?**	Kronos Magazine	16 (1-10)	Italy	Explore differences in perceived sources of stress, precompetitive appraisal, emotions, coping and motivation of referees depending on their perceived levels of support from refereeing organisations.	548 referees, mean age 22.46. Completed online survey and divided into three groups, low, medium or high levels of perceived support.	The results showed statistically significant differences between groups in terms of off-match stress, appraisal, emotions, task oriented coping, autonomous regulation and amotivation, highlighting the relationship between a supportive environment and positive outcomes such as less perceived stress, a challenge appraisal, positive emotions, task oriented coping strategies and intrinsic motivation. Thus, foster supportiveness policies by federations such as, helping on the conciliation between refereeing and life, transparency in promotion processes, and to provide resources to overcome the difficulties in refereeing could be a key element for the referees wellbeing
T. Webb, J. Cleland and J. O'Gorman	2017	**The Distribution of Power through a Media Campaign: The Respect Program, Referees, and Abuse in Association Football**	Journal of Global Sport Management	2 (162-181)	England	Review of the respect campaign 8 years after it launched across the UK and seeing the impact on officials.	2056 referees from across the UK completed an online survey about the impact of the Respect campaign and ongoing abuse.	Found often disenfranchised workforce who did not relate to the top levels and that despite the campaign introduction, continues to have ongoing issues with abuse, particularly verbal, at the grassroots level.

C. Castagna, M. Bizzini, S. D'Ottavio and S. C. Araujo Povoas	2018	**Sex Differences in Aerobic Fitness in Top-Class Soccer Referees**	Journal of strength and conditioning research	32 (3216-3221)	International	To compare the aerobic fitness level of male and female top-class Field Referees.	51 male referees on the 2014 list of candidates for 2014 World Cup and 40 female referees for 2015 Women's World Cup, all in first division locally and trained 3-6 times per week. Did treadmill test on 1% incline until exhaustion.	Data shows male matches played faster than female matches. Female players cover 2/3 the distance of males in the high intensity area. One study found female referees covered 10km with 1000m at high intensity. Women were younger 34 vs 38, shorter with lower BMI 21.28 vs 23.4. resting HR were similar. ON average females have a lower level of aerobic fitness. Only assessed aerobic fitness and not sprint ability or long sprint ability.
D. Castillo Alvira, J. Cámara Tobalina and J. Yanci Irigoyen	2018	**Influence of the maximum heart rate determination criterion on the quantification of the internal load in soccer refereeing**	Sports Medicine Archives	35 (228-235)	Spain	"The aims of this study were 1) to analyse the differences in internal load in official matches between on-field referees and assis- tant referees measured using different quantification methods, and 2) to find out if there are differences in the internal load using different criteria to determine individual HRmax (HRmax reached in an incremental text and HRmax reached in a match)".	41 referees from Spanish third division. 21 centre referees and 20 AR's. Age 26.95, BMI 23.38, experience 9.76 years. Determined internal match load by doing incremental field test the week before and comparing It to on field in match HR.	Studies have found that referees spend 95% of match time at >80% heart rate max. On field referees determined higher internal match loads than Assistant Referees. Study really comparing ways of assessing heart rate max in referees and whether they differ. They seem to differ in the output provided to responses.
Y Choi and J Roh	2018	**Activity profile and physiological responses of Korean amateur football referees during matches**	Journal of Physical Therapy Science	30 (351-354)	South Korea	Examining the physical requirements for amateur referees in Korean high school and college matches.	30 referees who officiated either the College cup competition or High School competition. GPS watch provided. Data divided between the high school and college referees, mean age approx. 37.5 with approx. 7y experience.	High school referees 6719m, mean speed 3.5km/h and max speed first half 23km/h and second half 22.3km/h. College was 7547m, mean speed 3.4km/h and max speed 23.7km/h and 23.4 km/h in first and second half. High school mean first half heart rate 150bpm and second half 148bpm, with max 177bpm and 175bpm. Distribution was 20% max, 30% hard, 24% mod, 17% light and 7% very light in first half - second half was 18%, 24%, 24%, 22% and 9% respectively. College referees was mean heart rate 146bpm and 145bpm in each half, max 169bpm and 170bpm, with heart rate distribution of 6%, 31%, 29% , 22%

								and 10% in first half and 6%, 29%, 29%, 22% and 12%.
D. R. N. Da Gama, R. A. M. Nunes, G. L. Guimarães, L. L. E Silva, J. B. P. De Castro and R. G. S. Vale	2018	**Analysis of the burnout levels of soccer referees working at amateur and professional leagues of rio de janeiro, brazil**	Journal of Physical Education and Sport	18(1168-1174)	Brazil	To analyze the components of this syndrome (physical and emotional exhaustion, depersonalization and reduction of the sense of professional achievement) in soccer referees working in amateur and professional league	36 referees from Rio in Brazil. 19 of which in the professional league in Rio. Used the Burnout Inventory for Referees (BIR). Uses Likert scale from 1-7 for the 3 domains of burnout as per the aim of the study. Also did sociodemographic questionnaire. Mean age 32.2 and experience was 7.5 years. Total 32 male referees and 4 females.	Those in the amateur league had higher scores in sport devaluation than professionals. Also scored worse with interpersonal component of burnout. Correlation between length of time refereeing and level of burnout related to the sport.
M. A. de Moura Simim, R. Melo Ferreira, M. V. Campos Souza, A. Custódio Marques and B. V. Correa da Silva	2018	**Psychological and social factors are subjective stress source in football referees**	Brazilian Futsal and Football Magazine	10 (475-480)	Brazil	To analyse the sources of stress in referees in Brazil after a match.	26 referees from the local league were given the TEPA stress survey post match.	Main stressors were; fields with no safety, lack of proper information about the competition, no payment, unprepared colleagues, delays, lack of recognition.
A. B. de Oliveira, E. N. Penna and D. A. Pires	2018	**Burnout syndrome in soccer referees**	Journal of Sports Psychology	27 (31-360	Brazil	Compare burnout over education, performance on the field, and how long they have been a referee for.	72 referees from 2 Brazilian state associations involved. Used the BIR.	Only findings was that only where the referees were from (which state) had an effect on perceived burnout.
D. R. N. D Gama, R. de Alkmim Moreira Nunes, G. L. Giumaraes, L. de Lima, E. Silva, J. B. P. de Castro and R. G. de Souza Vale	2018	**Analysis of the burnout levels of soccer referees working at amateur and professional leagues of Rio de Janeiro, Brazil**	Journal of Physical Education & Sport	18 (1168-1174)	Brazil	To understand the impact and effect of burnout on both amateur and professional league referees in Brazil	36 referees, 19 pro and 17 amateur took part. Used BIR and a sociodemographic questionnaire.	Showed professional referees referee more often, had more experience. Amateur referees felt more devaluation towards football the more they refereed, which wasn’t seen in pro referees. Referees In amateur league had higher burnout levels than in pro league.

G. S. Gillue, Y. R. Laloux, M. T. Alvarez and J. C. I. Feliu	2018	**Sources of Stress Inside and Outside the Match in Football Referees**	Physical Education and Sports notes	132 (22-31)	Spain	To identify causes of stress for referees both inside and outside the match and see if there is a relationship between experience and stress levels.	128 referees, 97% male, Mean age 26.02, range of experience from 2-22 years. In lower levels of Catalan football in Spain. Used the Sources of Officiating Stress questionnaire for in match stressors and then Questionnaire on Football Referees stress for out of match stressors. Each was from 1-7 with 7 highly agree with situation being stressful.	Refereeing generally is a highly stressful activity and can have negative impact on performance, satisfaction with the profession and decision to carry on refereeing. Most often cited sources of stress: fear of making mistakes, verbal abuse, threats to physical safety, interpersonal conflicts and time pressures. Social experiences outside of the match are as crucial in terms of wanting to continue refereeing. Experience in refereeing impacts how stressful things may seem. Less experience = more likely to be intimidated etc. Found high mean data (>4), for 10 scenarios, 6 in match and 4 out of match. Highest 2 were when a match turns out badly and getting injured (5.4 and 5.39 resp). Sources of stress outside the match mean 3.9 while inside the match were 3.6. Outside of the match the hardest were the refereeing committee politics and balancing refereeing with family life. Found as with other studies that verbal abuse wasn't stressful as referees saw it as part of their role and they internalise it. Greater the experience, the less stress inside the match perceived.
C. Jorge-Soto, F. Fernandez-Mendez, Z. Gonzalez-Gonzalez, F. G. Fandino-Reissmann, M. Otero-Agra, R. Barcala-Furelos, et al.	2018	**Football referees as first responders in cardiac arrest. Assessment of a basic life support training program**	Signa Vitae	14 (41-45)	Spain	To assess the CPR and AED use skills of amateur category football referees after a very brief training session.	35 amateur Spanish referees, 28 men, mean age 25. None had done BLS before. First did a brief questionnaire and then brief chain of survival theory session. Then finally all had an observed go at CPR.	24000 cardiac arrests in Spain per year with 5-7% survival rate. About half of OHCA seen by bystanders. Up to 2015 FIFA registered only 23 cardiac arrest deaths involved with the sport, 15 of which during a match. Mean 111 compressions per minute and 51.6m, 53.8% of total compressions were correct. Mean global quality was 73.3%. 16 did not pass the final exam with 13 of those putting pads in wrong location or modifying of the order of tasks. Only 1 of the referees knew

								what an AED was prior to the teaching and what it was for.
O. Kilic, U. Johnson, G. Kerkhoffs, P. Rosier and V. Gouttebarge	2018	**Exposure to physical and psychosocial stressors in relation to symptoms of common mental disorders among European professional football referees: a prospective cohort study**	BMJ Open Sport & Exercise Medicine	4 (e000306)	Europe	To investigate the association of physical and psychosocial stressors (eg injuries) with the one-season onset of symptoms of common Mental Disorders among European professional football referees.	Cohort study. Referees from a variety of European nations involved. Had to be professional referees. Asked about injuries, symptoms of CMD's. 292 completed the entire survey and 391 gave consent. Mean age 33. 7 years average in the professional leagues. 45% were referees, other 55% were AR's.	Prevalence ranged from 5.9% for CMD to 19% for eating disorders. More injuries and less social supports tied into the symptoms of CMD. Nearly half of all participants showed signs of CMD at baseline.
M. Campo and B. Louvet	2019	**Do high emotional intelligent soccer referees better cope with competitive stressors?**	Movement & Sport Sciences	105 (17-26)	French	Examine Emotional Intelligence in soccer referees and relationships with coping strategies	139 French referees, mean age 27.6, mean experience 11.4 - finally 116 male referees. 31% were at National or International level. Used trait EI questionnaire.	IDed 5 main stressors: time, lack of recognition, fear of physical harm, performance concerns and interpersonal conflicts. Need to be able to manage these things through Emotional Intelligence (EI). Note the inherent emotions of refereeing. This is the first study EI in sport officials. Choice of coping strategy is related to effective of the strategy itself. Positive correlation between self control and referee experience. Higher EI leads to better coping strategies and interpersonal emotional regulation.
C. Castagna, M. Bizzini, S. C. Araujo Povoas, K. Schenk, G. Busser and S. D'Ottavio	2019	**Aerobic Fitness in Top-Class Soccer Referees**	Journal of Strength & Conditioning Research	33 (3098-3104)	Italy	To examine the aerobic fitness of male top-class Field Referees and Assistant Referees.	52 referees male were the pre-selected ones for the 2014 World Cup (age 38.4 +/- 3.3, BMI 23.4). Had avg 7 years international experience. Trained 3-6 times per week. Run until exhaustion on 1% incline treadmill.	2 narrative reviews of referee physiology now. Cover more distance at higher intensity than players in EPL. AR's move 20-30% in sidewise movement. Resting HR 61, referees got to 97.8% of HR max and AR's got to 97.7%. Lrge difference in VO2 max between referees and AR's.

J. O. O. Chang Hwa and J. E. E. Haemi	2019	**Activity profiles of top-class players and referees and accuracy in foul decision-making during Korean National League soccer games**	Journal of Strength & Conditioning Research	33 (2530-2540)	South Korea	To perform match analysis and compare activity profiles of the elite-level soccer players and referees, analyze foul plays per player position and pitch location, and foul decision-making factors during nationally held professional league tournament in Korea.	Mostly focussed on players but also 14 referees mean age 37.5.	Cameras tracked referee movements, with 5 zones of activity: slow walking (0-6.9km/hr), walking (7-9.9km/h), jogging (10-12.9km/h), running (13-15.9km/h), high intensity running (16-17.9) and sprinting (18+). Avg distance for players was 11.9km and for the referees was 10.5km. More slow walking zone for referees than players. Players did more jogging and sprinting. Most reductions in distances were for both players and referees in the final 15 mins of the match.
V. L. da Silva, C. E. D. Depizzol, L. Carletti, R. L. Vancini, A. P. L. Leopoldo and A. S. Leopoldo	2019	**State of health and physical fitness of CBF (Brazilian Soccer Confederation) referees from the state of Espirito Santo**	Brazilian Journal of Sports Medicine	25 (147-151)	Brazil	To evaluate the health and physical fitness parameters of Brazilian Football Confederation (CBF) referees working in the state of Espírito Santo (capixabas referees).	16 referees from Espirito Santo, 87.5% men. Mean age 34.2, ages divided into 20-30 (12.5%), 30-40 (81.25%) and 40 plus (6.25%). Avg BMII 25.2.	Expend between 494 and 750kcal per match. 50% either needed improvement or had fair flexibility. Per the AHA cardiorespiratory conditions, 50% were good, 37.5% excellent and 12.5% fair. Suggestion that slight impairment in physical ability Assoc with age can be offset by experience in the game.
D. Fuente, E. Canton, F. Montes and M. A. S. Abella	2019	**Aggression towards Referees in Amateur Football in Spain: A Loglinear Approach**	Colombian Journal of Psychology	18 (1-18)	Spain	To detect the levels of abuse and misconduct that referees face in Spanish lower level football.	Looked at data from the Spanish Department of Sport who collate this kind of data. A record sheet with different agents of the match, the decision and the intensity of the response were recorded in 119 matches. The observer and the referee completed the sheet for each match.	Violence in sport is not the same as violence in the community so because we have a violent society doesn't mean we need to have a violent sport. According to Spanish data 93-98% of violent incidents in sport that were reported occurred in football. Out of 25 violent events that occurred in sport each year, 24 would be from football. However these incidents have decreased by almost 50% over the last decade from 2006-2016. In this same time period, referees were the victims of the abuse in 40-50% of events. About 100 cases per year involved police being called and this is stable over the 10 years. Aggression from each agent is independent of one another and not linked. Whereas the referee felt that this wasn't the case. The referees espeically felt the link between

								player and coach was the strongest.
E. Hong, Y. Jeong and P. Downward	2019	**Perceived organizational support, internal motivation, and work–family conflict among soccer referees**	Managing Sport & Leisure	24 (141-154)	South Korea	To assess the perceived levels of support that South Korean referees feel from their refereeing association and relationship with stressors.	Sent survey to stadia where national competitions were happening and 20-40 amateur referees would be staying. Total response was 260 completed surveys. 88.5% male, 58.6% married, avg age 33.69. All data collected on 7 point Likert scale.	Those that perceive support from their organisation tend to value it more. Internal motivation is where you do something because it makes you feel good or drives you. Work-family conflict is a common cause of issues across the globe and something that plays into refereeing. Referees felt more intrinsically motivated when they felt the referee association supported them. A high level of internal motivation decreased work-family conflict.
P. R. Santos-Silva, J. M. D'Andrea Greve, A. Pedrinelli, A. M. Almeida, B. B. Osorio, M. Ferreira, et al.	2019	**Comparing the Aerobic Fitness of Professional Male Soccer Players and Soccer Referees**	Current Sports Medicine Reports (Lippincott Williams & Wilkins)	18 (497-501)	Brazil	Comparing the fitness levels of players and referees in a Brazilian professional league.	50 male referees, and 61 male players included. Underwent clinical testing in the lab. Age avg 34.8 vs players 20.8.	Referee first appeared in 1863.Players aerobic fitness was 7.5% higher based on heart rate response to exercise and VO2 max. Overall showed very similar levels of fitness between the referees and players. Despite the sig older age of referees they didn't show the decline expected.
H. Aguirre-Loaiza, J. HolguÍn, J. Arenas, C. Nunezz, S. Barbosa-Granados and A. GarcÍa-Mas	2020	**Psychological characteristics of sports performance: analysis of professional and semiprofessional football referees**	Journal of Physical Education & Sport	20 (1861-1868)	Colombia			

I. Senecal, S. J. Howarth, G. D. Wells, I. Raymond and S. Mior	2020	**The Impact of Moderate and High Intensity Cardiovascular Exertion on Sub-Elite Soccer Referee's Cognitive Performance: A Lab-Based Study**	Journal of Sports Science and Medicine	20 (618-625)	Canada	To understand the effect of exercise exertion on cognitive performance as measured by the modified Stroop Color Word test (Stroop test) in sub-elite level soccer referees.	Pool of 12 grassroots referees in Ontario, 8 male, mean age 29.5, mean experience 14.3 years. Underwent 2 tests each within 5-10 days of each other. Used the Stroop colour test for decision making.	Cognitive performance improved with moderate intensity exercise. This improvement was retained following high intensity exercise too. Used VO2 max, lactate and heart rate to measure, they believe the second testing day may not have actually been high intensity exercise. Standardised the decision making but to something non refereeing so hard to compare to other studies.
T. Webb, M. Dicks, R. Thelwell, J. van der Kamp and G. Rix-Lievre	2020	**An analysis of soccer referee experiences in France and the Netherlands: Abuse, conflict, and level of support**	Sport Management Review	23 (52-65)	France, Netherlands	To compare the experiences, and support networks surrounding two national groups of soccer referees in France and the Netherlands.	Online survey sent to referees. 96,4% male form France and 98.8% male from Netherlands. Some professional referees down to grassroots.	English Football Association (FA) lost over 7,000 referees between 2011-2017. Some 42% have thought of leaving because of abuse and 20% due to lack of support. 68.1% of sampled French officials had experienced verbal abuse, 51% in Netherlands. 16% of French referees physically abused and 14.6% of Dutch referees. Referees felt abuse was worse at lower levels than higher levels. 21.6% of French and 11.7% of Dutch were considering leaving in the next 12 months. Some thoughts from referees about how to help, stronger disciplinary proceedings against aggressors, more support from federations, there were differences in support felt pending the level they refereed at. Some other ideas include conflict resolution training,
J. L. Aguilar, A. Castillo-Rodriguez, J. L. Chinchilla-Minguee and W. Onetti-Onetti	2021	**Relationship between age, category and experience with the soccer referee's self-efficacy**	Peerj	9 (16)	Spain	To show that self efficacy is higher in more advanced referees.	REFS (Referee Self Efficacy Scale) to measure self efficacy. 256 male referees from Andalusia (72 to National level), between 18-34 yo.	5 million referees around the world. Self efficacy higher in referees of higher quality.
A. Castillo-Rodriguez, J. Lopez-Aguilar and I. Alonso-Arbiol	2021	**Relationship Between Physical-Physiological and Psychological Responses in Amateur Soccer Referees**	Sports Psychology Magazine	30 (26/37)	Spain	Aims to understand physical, physiological and psychological responses and interrelations in amateur football matches.	15 referees from Andalusian association were included. 21 matches were included in the data.	More anxiety and less self confidence and stress control and motivation in match versus pre season testing. Stress control went down when heart rate got above 80% of maximal.

J. Devís-Devís, J. Serrano-Durá and P. Molina	2021	**"The Referee Plays to Be Insulted!": An Exploratory Qualitative Study on the Spanish Football Referees' Experiences of Aggression, Violence, and Coping**	Frontiers in Psychology	12 (1-12)	Spain	To know the types of aggression and violence referees experienced, and the strategies they used to manage these behaviours and to understand the personal and contextual characteristics involved in the aggression, violence, and coping experienced by Spanish football referees.	4 male and 4 female referees aged between 19 and 34 with 2-17 years of experience involved. Used interviews to answer questions.	Aggression is a consequence of frustration. Violence is a physical form of aggression. Younger referees more exposed than older referees. One study found 15% of Swedish officials had experienced some form of physical violence. Studies from Aus and Sweden found referees confronted players like this by asking them to restore their conduct to normal. Most frequent hostile reaction was verbal abuse - usually foul language. Sexist comments came from older people towards the female referees. Also episodes of female spectators hurling sexist abuse at female referees - they take on the macho environment around them. From male players to female referees tended to be in the way of social media after the match - adding as a friend etc. Some normalised the verbal abuse stating if it happened at the match it's better than happening at home with DV. 4 of the referees had experienced physical violence. Also noted aggressive environments in the stands with fights among spectators could lead to violence towards the referee. A vast array of coping strategies was highlighted.
P. Gorczynski and T. Webb	2021	**Developing a mental health research agenda for football referees**	Soccer & Society	22 (655-662)	England	To outline the importance of why an epidemiological understanding of mental health symptoms and disorders amongst referees is necessary and desperately needed.	Reviewing the existing scaffolds that exist and planning for integrations of new ones.	Prevalence - rates in a time period. Incidence is new cases in a time period. Refers to Gouttebarge et al which is coming up below. Want to develop a framework to understand the burden of mental health issues in the refereeing community using the behavioural epidemiology framework.

A. Marchante and R. Cervigon	2021	**Assessment of Physiological and Psychological Factors in Nonprofessional Football Referees**	International Journal of Psychophysiology	168 (S138)	Spain	Analyse the technical, physical and physiological factors that impact on football referees able to achieve a good match performance.	12 referees from Cuenca, Spain. Mean age 22.67. Average years experience was 6 yrs. Assessed heart rate, physical activity through fitness testing and psychology through in-person interview.	No differences between heart rate in referees and negative correlation between heart rate and concentration. Heart rate minimum showed negative correlation with hostility and depression.
M. L. Martín-Sánchez, M. Marín-Farrona, L. Gallardo, J. García-Unanue, Á. Matute-Llorente, J. A. Casajús, et al.	2021	**Level of studies and experience of female football referees and assistant referees in Spain: Women's First Division**	Culture, Science and Sports	16 (653-660)	Spain	The objective of this research was to analyze the sociodemographic characteristics of the referees and assistant referees belonging to the first division of women's football in Spain.	17 referees and 28 Assistant Referees. Average age 28.1, in the Spanish first division competition, all female.	53% completed university as referees and 43% in Assistant Referees. 41% completed masters as referees and 36% as Assistant Referees. 6% completed professional training as referees and 14% as Assistant Referees. Average 12.4 years as referees, Assistant Referees 8.8 years. Referees trained 4-5 times per week while Assistant Referees trained 4 times per week. Those who had FIFA badges had been refereed for avg 13 years compared to those not with FIFA of 8.8 years.
V. Moreno-Perez, M. L. Martín-Sánchez, J. Del Coso, J. L. Felipe, J. Courel-Ibañez and J. Sánchez-Sánchez	2021	**Impact of COVID-19 lockdown on match activity and physical performance in professional football referees**	Biology of Sport	38 (761-765)	Spain	The aim of this study was to investigate the effect of COVID-19 lockdown on match-play metrics in professional football referees during official matches of the Spanish professional leagues.	42 professional referees from Spain, mean age 37.8. GPS units used to track movement and heart rate. Covered in different areas; total distance, <6km/hr, 6-12km/hr, 12-18km/hr, 18-21km/hr and 21-24km/hr and greater than 24km/hr.	COVID lockdowns of 8 weeks in Spain meant the supervisory body created tailored programs which could be completed from home. The lockdown showed decrease in total distance covered, distance covered at all speeds >6km/h, peak acceleration and deceleration, distance and number of sprints, and avg heart rate. But saw more distance at the <6km/hr range. Shows covered more distance at lower speeds in attempt to cover the distance still. Had 8 weeks of detraining at home with 4 weeks outside once lockdown lifted but data suggests this wasn't enough to cover the detraining.

N. Orviz-Martinez, M. Botey-Fullat and S. Arce-Garcia	2021	**Analysis of Burnout and Psychosocial Factors in Grassroot Football Referees**	International Journal of Environmental Research and Public Health	18 (1-18)	Spain	Examine whether violence and the environment play a role in burnout.	203 Spanish referees completed a survey, 94% men, avg age 23.5 and all refereed grassroots football. Most had experience in the 5-10 year range. Questionnaire provided as designed by the team.	Burnout is prolonged response to chronic stress. Emotional exhaustion can decrease productivity or correct calls but can also devalue the role in the sport. The environment and the level of verbal and physical absue increase emotional exhaustion and burnout. 23-24 year olds had the highest values in all domains except effectiveness, therefore being the more prone to burnout. Whereas the 14-18 year olds had the lowest value in all domains and the highest in effectiveness.
E. Özdamar, S. Hazir Aytar and A. Kin İŞler	2021	**Investigation of the physiological load of football local referees during competition according to league level**	Journal of Physical Education & Sports Sciences	19 (29-38)	Turkey	Determine the physiological load for referees at the local level.	31 male referees, mean age 25.3. Heart rate and RPE collected from officials. Did a Yo-Yo for maximal heart rate assessment.	Maximal heart rate in first and second halves were 80% and 79.9% respectively. Mean RPE was 11.4 and 11.3 for each half.
R. Rebelo-Gonçalves, H. Pardal, L. Coelho, R. Antunes and N. Amaro	2021	**Physiological and mechanical loads in Portuguese sub-elite football refereeing - a preliminary study**	Sports Psychology Notebooks	21 (213-223)	Portugal	To examine the variation between-half in physiological and mechanical load parameters in sub-elite referees and according to their specific role as officials	3 officials. All male, 1 centre referee and 2 Assistant Referees. Age 32 for centre and 20.5 for AR's. Wore a bioharness for HR etc.	External and internal load emerged in 2004. External is work done by the athlete and internal load is the psychophysiological response to the work. No differences in work were noted between halves or between periods of the match. There was a sig difference in output from referee vs Assistant referees.
A. Urhausen, J. P. Vivas, C. Lambert and B. Weiler	2022	**Cardiovascular Stress in Football Referees.**	German Journal of Sports Medicine	73 (30-340	Luxembourg	To study cardiovascular health including risk-factors and evaluate the stress (physical and psychological).	21 referees from 2 upper division of Luxembourg (9 FIFA, 14 non-FIFA). Received 3-lead Holter before, during and after a match. One match per referee.	Age 36 +/- 8 years, 17 had at least one cardiovascular risk factor, 2 had 4 risk factors, 2 smokers. Stress showed sig increase in heart rate (16 +/- 10bpm). Note 11.2km +/- 1.05km. Mean heart rate 154bpm.
C. M. Arjona, B. R. Fernandes, T. D. Dos Santos, W. O. Onetti and A. C. Rodriguez	2022	**Effect of the soccer referee's experience in sports competition**	Culture, Science and Sports	17 (41-51)	Portugal, Spain	To analyse semi-professional football referees and variables including, psychological, physiological and compare relative to experience.	Questionnaire sent out to football referees in Portugal and Spain to understand levels of psychological stress during matches.	153 referees mean age 23.2y, more experience showed less negative psychology and more positive psychology.

A. Castillo-Rodríguez, C. Muñoz-Arjona and W. Onetti-Onetti	2022	**National vs. Non-National Soccer Referee: Physiological, Physical, and Psychological Characteristics**	Research Quarterly for Exercise & Sport	93 (804-812)	Spain	To analyse the variables: psychology (self esteem, anxiety) pre competition and physical and physiological changes during a match between national and non-national referees.	153 referees, mean age 23.2, mean experience 6 years. Did CSAI-2 and Rosenberg questionnaire before the match. And GPS data for during the match.	National referees had higher self esteem and self-confidence compared to non-national referees. National referees had higher mean and max heart rates. Some correlation with age and experience with self-esteem and self confidence which may explain up to 20% of the variance but not all.
P. Dawson, T. Webb and P. Downward	2022	**Abuse is not a zero-sum game! The case for zero tolerance of match official physical and verbal abuse**	European Journal of Sport Science	22 (417-424)	France, Netherlands	Examine the physical and verbal abuse of referees and the relationship between abuse and their expressed intentions to leave the sport.	Online questionnaires to referees in France and the Netherlands. 3% and 1% female respectively. Majority had 3-5 years experience in France and 6-10 years in Netherlands.	National survey in the USA with sport officials generally, 87% had experienced verbal abuse. Organisational support is crucial to retention. 70% experienced verbal abuse in France and 50% in the Netherlands. 17% experienced physical abuse in France and 15% in Netherlands. 22% have thought of leaving in the next 12 months in France and 12% in the Netherlands. 40% and 20% feel intimidated when refereeing certain teams. There is a theoretical link between verbal and physical violence in the game. In France, female referees faced less abuse than males. Suggestion that abuse happens when it is less costly than the outcome and in amateur football there is easy 'access' to officials and also able to see it in the media etc.
X. E. Fernandez, M. Priego-Ojeda, A. R. Morente and C. A. Mora	2022	**Relationship between emotional intelligence, burnout and health perception in a sample of football Spanish referees**	Challenges-New Trends in Physical Education, Sports and Recreation	44 (960-975)	Spain	To evaluate the relationship between the Emotional Intelligence of football referees with subjective perceptions of health.	4099 Spanish referees aged 14-66, 92% men. Used the trait Meta-Mood Scale (TMMS) for EI assessment. Emotional and general health using a 12 item General Health Questionnaire. Used Oldenburg Burnout Inventory.	See burnout having a positive effect rate on health problems but also on perceived health awareness. Those who had higher scores of attention had stronger relationship between burnout and health problems. Emotional Intelligence is a resource that lessens the negative effects of stress and burnout. Other studies found more years referee equalled less stress experienced. Found those age 38-43y had best perception of health with lowest amount of burnout.

M. Ferreira, C. Ferreira and L. C. de Abreu	2022	**Risk of cardiovascular disease in soccer referees: a cross sectional study**	Journal of Human Growth and Development	32 (298-308)	Brazil	To evaluate the cardiovascular risk of high performance soccer referees.	50 Brazilian referees studied at a pre season camp. Split the group into <35yo and >35yo. Examined a range of factors with specific details of inclusion of each included in the paper but not included here. Used the Framingham and PROCAM models to predict cardiovascular risk in the next 10 years. Mean age 34.8 with BMI of 23.59. Examined biochem, history, exam and echocardiogram.	Most common abnormalities were sinus bradycardia and incomplete RBBB. Most reference values for biochem were within normal range on average. Had a normal mean blood pressure. Stat significant difference in Framingham risk in the next 10 years for the over 35 year olds compared to under 35 yo. On the Framingham risk only 2 individuals had a risk moderate to high.
P. Gorczynski and R. Thelwell	2022	**Examining mental health literacy, depressive symptoms, help seeking behaviors, and wellbeing in soccer match officials in the UK**	International Journal of Sport Psychology	53 (25-35)	England	As such the purpose of this study was to examine the levels of mental health literacy, depressive symptoms, wellbeing, and help seeking behaviours of soccer match officials in the UK.	45 female and 268 male referees from the UK took part via a Google Doc link. Mean age 27.4 years. 53% refereed 1-3 hours per week. Used Mental health literacy scale, higher score is better literacy. CESD-R scale for depressive symptoms, higher score, greater severity of depression. General help seeking questionnaire for seek help for mental health issues. WEMWBS is mental wellbeing score.	Match officials have been killed eg. Jose Valdemar Hernandez Capetillo, Otávio Jordão da Silva, Richard Nieuwenhuizen, Ricardo Portillo and some have killed themselves (e.g., Babak Rafati). Mean score for MHLS was 98. Females sig higher score than males. MHLS scores higher for those refereeing 1-3 hours per week vs 4+ hours. 2% indicated symptoms of Major Depressive Disorder, 7% for probable depressive disorder. Most likely to seek help from an intimate partner and mental health professional. Males had sig higher WEMWBS score than females. Had lower MHLS scores than in other sporting cohorts. Only 1.8% indicated may have had symptoms related to a depressive disorder which is lower than other quoted cohorts. Those that were lesbian or bisexual also had lower levels of wellbeing and more symptoms.

Z. Y. Liu, L. Y. Zhao, S. Z. Wang, Y. B. Gao and L. G. Zhang	2022	**The Association between Occupational Stress and Mental Health among Chinese Soccer Referees in the Early Stage of Reopening Soccer Matches during the COVID-19 Pandemic Outbreak: A Moderated Mediation Model**	International Journal of Environmental Research and Public Health	19 (1-16)	China	To investigate the mediating role of job burnout in the association between occupational stress and mental health of soccer referees.	317 Chinese referees completed the survey during the open time. 54.1% male, age range 19-45. Used Effort-Reward Imbalance scale for occupational stress. Used DAS-21 for depression/anxiety. Used MBI-GS for burnout levels. Used the PSSS for perceived social support levels.	Excessive time pressures, impact on travel and support all tie into the work stressors that exist for referees. Burnout has known serious risks attached to it. Mild or above stress was 5.9%, mild or above anxiety was 22.7% and mild or above depressive symptoms was 16.1%. Occupational stress inversely related to social support but positively related to burnout and depression, anxiety and pressure. Occupational stress positively correlated with poor mental health conditions. Job burnout negatively associated with mental health.
J. Marin-Montin and P. Bianchi	2022	**"Go and Wash up!": Gender Violence in Female Refereeing and Its Media Coverage in Brazil and Spain**	Communication & Sport	0 (1-25)	Brazil, Spain	Analyse how the Brazilian and Spanish media cover female football referees and specifically the focus on violence against female officials.	Two styles of data collection, trawling the online and TV data from 2 main sites in each country and also a focus group in each country with female referees.	Combination of both being in a profession with minimal support but also having to face their own gender stereotypes. One reason they drop out is motherhood, with difficulty balancing both together. But other studies also found lack of respect from male counterparts, perceived inequalities and suffering gender abuse. Pay differences, women earned 95% less in top Spanish women's comp than the equivalent men. Media portrayal doesn't help either. Widespread comments in the media, some supportive and some derogatory don't help the situation. In Brazil, significant data shows it is a violent sport towards officials at all levels and this is still the case with female officials. 33 media reports were covered in the time frame. Sig amounts of sexist comments with quotes throughout the paper. 2 reports of physical violence against different female referees. Statements in the media ranged from sexist abuse, covering of abuse cases, female referee groups standing up for themselves, statements by families of female referees, comments from fans at

								the matches involved, coach eyewitness statements. Focus groups: sexist comments were the most common type of abuse, mention that sanctions on clubs were weak but that when made public and tarnished the image of the club, they acted more. 4 in each group of 7 said the abuse had made them reconsider whether to continue refereeing. All agreed the media are covering it more but with a more morbid curiosity. Aggression diminished with the presence of TV cameras. 'Go and wash up' was the most common single abuse item. Only visible types of abuse were covered in the media, not the subtle stuff.
M. L. Martin-Sanchez, J. M. Oliva-Lozano, J. Garcia-Unanue, J. L. Felipe, V. Moreno-Pérez, L. Gallardo, et al.	2022	**Physical demands in Spanish male and female elite football referees during the competition: a prospective observational study**	Science & Medicine in Football	6 (566-571)	Spain	Examine the physical demands of male and female elite football referees and the differences that may exist.	19 male and 17 female referees, during 409 matches. Used GPS tracking.	Men experienced sig more physical stress in their league compared to women in female league in regards to total distance, explosive distance, HIA, total sprints, sprint distance, HIA distance, max speed, total acceleration/deceleration.

D. Nogueira, L. Fontes, A. R. Gomes and R. Resende	2022	**Referees' Emotions and Performance Perception: The Importance of Stress and Cognitive Appraisal.**	Sports Psychology Notebooks	22 (156-170)	Portugal	To understand how football referees adapt to stress, considering, in an integrated manner, the stress factors, the cognitive appraisal processes, the emotional reactions, and their performance perception.	Stress tends to come when demands of the environment exceed their perceived levels of coping. Pre match experienced stress related to decision making and sports conditioning. Most emotions pre match were happiness and excitement. Stress related to errors, sports condition were related to higher anxiety. Have high levels of coping perception and low levels of threat perception. Happiness and excitement first followed by anxiety. Less experience at the top level increases anxiety and anger. Stressors were predictive of negative emotions.	Stress tends to come when demands of the environment exceed their perceived levels of coping. Pre match experienced stress related to decision making and sports conditioning. Most emotions pre match were happiness and excitement. Stress related to errors, sports condition were related to higher anxiety. Have high levels of coping perception and low levels of threat perception. Happiness and excitement first followed by anxiety. Less experience at the top level increases anxiety and anger. Stressors were predictive of negative emotions.
E. Ozaeta, U. Fernández-Lasa, I. Martínez-Aldama, R. Cayero and D. Castillo	2022	**Match Physical and Physiological Response of Amateur Soccer Referees: A Comparison between Halves and Match Periods**	International Journal of Environmental Research and Public Health	19 (1-14)	Spain	To describe the differences in external and internal load in amateur match officials (Field Referees and Assistant Referees) between the 1st and 2nd half, and to analyze the differences among 15 min periods of the match (0–15 min, 15–30 min, 30–45 min, 45–60 min, 60–75 min and 75–90 min).	69 Spanish referees. 23 centre referees age 25.65 and 46 Assistant Referees mean age 23.11. 23 matches were recorded on video. External load was distance and speed. Internal load was heart rate by monitor.	Centre referees covered 66% as much distance as Assistant Referees at high intensity, so less bursts of sprint for referees. Mean heart rate for referees is 85% of mac and 77% for Assistant Referees. For centre referees, differences in cadence, speed and power 1st half to second half but no difference in distance covered. Referees covered more distance in the final 15 mins than any other block. There were some minor differences in the 15 min blocks for distances covered and time spent in energy zones. No external load differences for Assistant Referees between halves, had lower heart rate mean in the second half. The changes in 15 min periods and activity likely from changes in match intensity.

P. A. Potrac, E. T. Hall and A. J. Nichol	2022	**Fear, Anger, and Loneliness: Emotional Pain and Referee Attrition in English Grassroots Football**	Sociology of Sport Journal	39 (298-308)	England	Examine the emotional pain felt by ex-referees and how their relationships with other participants in football became so bad they needed to leave.	Online survey and interview with 251 former referees, 236 male form England.	Identified 2 areas in USA basketball where referees leave - the retention point (leave due to social supports, not enjoying the game, no mentors etc) or because of lack of promotion and moving up the ranks. All highlighted their quitting was due to emotional pain from other participants in the sport. Often encountered match day encounters as abusive, received verbal and physical abuse. Sense of anxiety due to not knowing what they would face on matchday. Inadequate support from the associations left feelings of betrayal, felt alone in disciplinary issues as they didn't have the support they would have expected. Felt prepared to know the laws of the game but not how to interact with the other stakeholders and manage those battles. Felt like they were ignored by the association - request for decreased schedules due to injury etc were ignored. Mentors played a big role in the process, good ones kept referees and bad ones scared them away.
M. L. M. Sánchez, J. M. Oliva-Lozano, J. García-Unanue, P. Krustrup, J. L. Felipe, V. Moreno-Pérez, et al.	2022	**Association between Fitness Level and Physical Match Demands of Professional Female Football Referees**	International Journal of Environmental Research and Public Health	19 (1-10)	Spain	To analyse the association between the physical performance in a match and the fitness level of elite female football referees in order to specifically guide female football referees' training	17 female referees, avg age 29, avg 4 years in top Spanish league and at least 10 years refereeing. Trained on average 4 times a week. Undertook the repeated sprint test and Yo-Yo test. And then observed matches as well.	Previous studies have found lower levels of physical activity in female referees compared to male counterparts. Demands were sig lower in second half and so was distance covered, max speed, explosive distance. Found that the fitness tests correlated with ability to referee and fitness in match. Lower total distances covered that studies in men. Didn't cover any internal load variables.

J. Wesolowska, A. Jurczak, S. Wieder-Huszla, K. Jarosz, A. Jurewicz and M. Marchlewicz	2022	**Comparison of the risk of cardiovascular diseases, stroke, and diabetes among the selected group of football referees and the group of general population men from Northern Poland - a pilot study**	European Review for Medical and Pharmacological Sciences	26 (3151-3160)	Poland	Comparing body fat and visceral adiposity levels between football referees and a control group as well as assessment of risk of diabetes, stroke and cardiovascular disease in the referee cohort.	56 male football referees from Poland, mean age 32. And control group of Polish men, mean age 47. Did a health questionnaire, personality questionnaire, measured visceral body fat.	The non-referees had higher BMI, trunk fat, visceral adipose, total body fat and excessively developed adipose. Referees had higher waist to hip ratio, more no adipose mass and body water. Both groups had similar levels of health oriented activity. Referees were less conscientious than non referees. Both groups had similar levels of self reported health. Referees had sig less arterial hypertension, and sig more with no illness but no other changes in risk factors. Had sig less smokers. Had sig less people dieting/limiting certain food types vs the regular controls. Had sig less people never or almost never having alcohol than the controls.
J. F. da Silva, A. S. Teixeira, J. de Carvalho, P. D. Salvador, C. Castagna, A. P. Ventura, et al.	2023	**Match activity profile and heart rate responses of top-level soccer referees during Brazilian national first and second division and regional championships**	Science and Medicine in Football	7 (263-271)	Brazil	To examine the differences in heart rate between different divisions and between referee and Assistant Referees in Brazilian leagues.	51 referees and 87 Assistant Referees. Used GPS and heart rate monitor for match activities.	Referees and Assistant Referees covered more distance at higher intensity in the top 2 divisions compared with the regional division of Brazil. Saw sig differences in total distance, HIA and sprinting between halves. Average heart rate max increased throughout the match.
Y. Lima, S. Devran, N. D. Oz, T. Webb and B. Bayraktar	2022	**Examining the mental health status of referees in the Turkish professional football league**	Science and Medicine in Football	7 (272-278)	Turkey	To understand the mental health status of referees in the Turkish professional league.	Online survey sent to all referees who officiate the professional leagues. 433 respondents.	Referees 18-27 yo had sig higher anxiety, depression and stress than referees >38yo. Same things were higher in single compared to married referees. Lower level referees had higher levels of all things than those at the higher levels. Higher scores in all these things were associated with lower income, worse injury history and worse match performance.

G. Martinez-Torremocha, M. L. Martin-Sanchez, J. Garcia-Unanue, J. L. Felipe, V. Moreno-Perez, V. Paredes-Hernandez, et al.	2022	**Physical demands on professional Spanish football referees during matches**	Science and Medicine in Football	7 (139-145)	Spain	To analyse the external and internal load of professional Spanish football referees during matches, and to compare the physical demands between halves and between referees in different categories.	40 professional referees from Spanish top and second divisions. Mean age 35.88, 19 in top division and 21 in second division. All had at least 10 years of experience. All trained at least 5 times per week, and refereed one match per weekend. Tracked distance using a GPS. Created 6 zones of movement. Had heart rate monitor for internal match loads. Total 419 matches recorded.	Again reinforced the 10-12km distance covered information and the 85-90% of heart rate max. Lower mean heart rate in Division 1 referees. Nil other significant differences in other variables. Total distance covered was similar across divisions. Second Division referees covered more at 12-18km/hr and 18-21km/hr zones. Both had decreased average heart rates in second halves.
